# Revision of the genus *Exaesiopus* Reichardt, 1926 (Coleoptera, Histeridae, Saprininae)

**DOI:** 10.3897/zookeys.479.8738

**Published:** 2015-01-29

**Authors:** Tomáš Lackner

**Affiliations:** 1 Czech University of Life Sciences, Faculty of Forestry and Wood Sciences, Department of Forest Protection and Entomology, Kamýcká 1176, CZ-165 21 Praha 6 – Suchdol, Czech Republic

**Keywords:** *Exaesiopus*, revision, Coleoptera, Histeridae, Saprininae, Palaearctic and Afrotropical Regions

## Abstract

The genus *Exaesiopus* Reichardt, 1926 is revised herein. It now contains seven species; one new combination is proposed: *Pachylopus glaucus* = *Exaesiopus glaucus* (Bickhardt, 1914), **comb. n.**, and one species is described as new: *Exaesiopus therondi* **sp. n.** from Afghanistan. Subspecies *Exaesiopus grossipes berberus* Peyerimhoff, 1936 is sunk in synonymy with *Exaesiopus grossipes* (Marseul, 1855), **syn. n.** Lectotypes and paralectotypes, respectively, for *Saprinus grossipes* Marseul, 1855, *Exaesiopus grossipes berberus* Peyerimhoff, 1936 and a neotype for *Pachylopus glaucus* Bickhardt, 1914 are designated. *Exaesiopus grossipes* is re-described; other species are provided with diagnostic descriptions and supplemented by SEM micrographs, colour images, and line drawings of their male genitalia. A key to species is given. *Exaesiopus glaucus* (Bickhardt, 1914) is newly recorded from the Republic of South Africa; *Exaesiopus torvus* Reichardt, 1926 is new to Uzbekistan and Russia; *Exaesiopus atrovirens* Reichardt, 1926 is new to Ukraine and Tajikistan; and *Exaesiopus henoni* (Schmidt, 1896) is new to Libya and Djibouti.

## Introduction

The genus *Exaesiopus* was erected by Reichardt (1926) based on the species *Saprinus grossipes* Marseul, 1855. Reichardt (1926) mainly used the presence of prosternal vestiture as the discriminating character from the presumably closely related genus *Hypocaccus* C. Thomson, 1867. In the same paper he described two further species from ex-Soviet Middle Asia, *Exaesiopus torvus* and *Exaesiopus atrovirens*, and combined the species *Pachylopus henoni* Schmidt, 1896 into *Exaesiopus*. Peyerimhoff (1936), based on the elytral punctation, split the species *Exaesiopus grossipes* into two subspecies: *Exaesiopus grossipes grossipes* from the northern shore of the Mediterranean Sea and South Europe, and *Exaesiopus grossipes berberus* from North Africa (Algeria, Tunisia). Thérond (1964) added an additional species, *Exaesiopus laevis* from Somalia, to the genus. Lackner (2010) included a diagnosis and a brief discussion of the monophyly of *Exaesiopus* in his Review of the Palaearctic Genera of Saprininae (Histeridae), without having examined the Somali species. In the discussion pertaining to *Exaesiopus* I mentioned that the genus is most likely non-monophyletic and its relationship to the genus *Hypocaccus* should be the focus of future studies. The single synapomorphy of Old World *Exaesiopus* (ciliate pronotal hypomeron) is prone to parallelism and shared with at least two Nearctic species of *Hypocaccus*. The present revision of the genus is therefore meant to act chiefly as a tool for identifying *Hypocaccus*-like Saprininae species with ciliate pleura and sterna from the Old World. These are grouped here under the currently valid genus *Exaesiopus*; however, a worldwide review and phylogenetic study of all *Hypocaccus*-like taxa is a prerequisite for a sound classification of this difficult group of beetles. During the years 2009–2014 I have had the opportunity to examine many rare Saprininae taxa, including *Exaesiopus laevis* from Somalia and Hypocaccus (Hypocaccus) glaucus (Bickhardt) from Namibia. The results of these examinations are presented below. This work presents another contribution to on-going revisionary work on the genera of the subfamily Saprininae (Lackner 2009a, b, c, 2010, 2011a, b; Lackner 2012; Lackner 2013a, b; Lackner and Gomy 2013; Lackner 2014a, b, c, d; Lackner and Tishechkin 2014; Tishechkin and Lackner 2012).

## Material and methods

All dry-mounted specimens were relaxed in warm water for several hours or overnight, depending on the body size. After removal from their original cards, the beetles were side-mounted on triangular points and observed under a Nikon 102 stereoscopic microscope with diffused light. Some structures were studied using methods described by Ôhara (1994): the head and male genitalia were macerated in a hot 10% KOH solution for about 15 minutes, cleared in 80% alcohol, macerated in lactic acid with fuchsine, incubated at 60 °C for two hours, and subsequently transferred into a mixture of glacial acetic acid 1 part and methyl salicylate 1 part heated at 60 °C for 15 minutes and cleared in xylene. Specimens were then observed in α-terpineol in a small glass dish. Digital photographs of the male terminalia, mouthparts and antenna were taken by a Nikon 4500 Coolpix camera and edited in Adobe Photoshop CS4. Based on the photographs or direct observations, the genitalia were drawn using a light-box Hakuba klv-7000. SEM photographs were taken with a JSM 6301F microscope at the laboratory of Faculty of Agriculture, Hokkaido University, Sapporo, Japan as well as at the Laboratory of the Electron Microscopy at the Faculty of Biology, Charles University, Prague, Czech Republic. Colour images were produced by F. Slamka (Bratislava, Slovakia). All available specimens were measured with an ocular micrometre. Beetle terminology follows that of Ôhara (1994) and Lackner (2010). Separate lines of the same label are demarcated by a slash (/). The following acronyms of museums and private collections are used throughout the text:

CAS Collection Alexander Sokolov, Moscow, Russia;

CYG Collection Yves Gomy, Nevers, France;

NHM The Natural History Museum, London, United Kingdom (R. Booth);

MNHN Muséum National d’Histoire Naturelle, Paris, France (A. Taghavian);

ZMHUB Museum für Naturkunde, Leibnitz Geselschaft, Berlin, Germany (B. Jaeger);

MSNM Museo Civico di Storia Naturale, Milano, Italy (F. Rigato);

NCB Naturalis Biodiversity Centre, Leiden, Netherlands (B. Brugge);

TLAN Tomáš Lackner collection, temporarily housed at Naturalis Biodiversity Centre, Leiden, Netherlands;

TMSA Transvaal Museum of Natural History, Pretoria, Republic of South Africa (R. Müller);

ZIN Zoological Institute of the Russian Academy of Sciences, St. Petersburg, Russia (B. Kataev).

Abbreviations of morphological measurements follow Ôhara (1994) and are used throughout the text as follows:

APW Width between anterior angles of pronotum

EL Length of elytron along sutural line

EW Maximal width between outer margins of elytra

PEL Length between anterior angles of pronotum and apices of elytra

PPW Width between posterior angles of pronotum.

## Taxonomy

### Exaesiopus

Taxon classificationAnimaliaColeopteraHisteridae

Reichardt, 1926

Exaesiopus Reichardt 1926: 14. Type species *Saprinus grossipes* Marseul, 1855, original designation.
Exaesiopus : Reichardt (1941): 156, 329; Peyerimhoff (1936): 226; Kryzhanovskij and Reichardt (1976): 112, 232; Mazur and Kaszab (1980): 7, 61; Vienna (1980): 117, 195; Mazur (1984): 101; Mazur (1997): 263; Yélamos (2002): 245, 338; Mazur (2004): 92; Lackner (2010): 63, 111; Mazur (2011): 210.

#### Diagnosis.

Although the genus has been recently diagnosed (Lackner 2010: 111), it requires modification to accommodate the newly examined species *Exaesiopus laevis*, the newly included *Exaesiopus glaucus* (Bickhardt), and the newly described *Exaesiopus therondi*. Body in most species strongly convex, especially dorsally; cuticle light to dark brown to almost black, in several species with (feeble) green lustre. Clypeus anteriorly elevated (Fig. 4); frontal stria carinate (Fig. 2); frons with several chevrons, occasionally surrounded by numerous tiny rugae (Fig. 2); pronotal hypomeron setose (Fig. 55). Elytra in most species with punctation; in all species striate; pleura and sterna furnished with short setae (Fig. 41). Prosternum with both sets of striae complete, and occasionally with weakly impressed prosternal foveae (Fig. 6). Protibia with 2–3 (large) teeth topped by triangular denticle; protibial spur in most species inconspicuous (apparently absent); metafemora thickened; metatibiae triangularly dilated and thickened (except for *Exaesiopus glaucus*).

#### Differential diagnosis.

Members of *Exaesiopus* are generally morphologically most similar to the Old World species of the genus *Hypocaccus*, differing from them chiefly by the setose pronotal hypomeron, strongly convex body, thickened metafemora and triangularly dilated and thickened metatibiae. In North America, however, there are at least two species of *Hypocaccus* (*Hypocaccus propensus* (Casey, 1893) and *Hypocaccus servilis* Casey, 1893) that are characterized by the presence of hypomeral setae.

#### Biology.

*Exaesiopus* species are almost exclusively found in sandy soils, beach dunes, river sands, and are also found in sandy areas further inland (e.g. Sahara desert). Morphologically they are well adapted to their fossorial habits. Species are often collected on rotting biological matter, e.g. under faeces, dead fish etc., and are occasionally found under coastal wrack or by shore washing. The middle Asian *Exaesiopus atrovirens* and *Exaesiopus torvus* are sometimes found burrowing under *Tamarix*. The biology of *Exaesiopus laevis* and *Exaesiopus therondi* is unknown, the latter has been found inside the stomach of Kentish plover (*Charadrius alexandrinus* L. (Aves)).

#### Distribution.

Genus *Exaesiopus* has a generally circum-Mediterranean-Caspian-Turanian distribution, most westerly occurring on the Canary Islands, reaching Afghanistan in the east. Its members have also been collected in the Sahara desert (Laghouat, Algeria), reaching as far east as northern Somalia (*Exaesiopus laevis*) or Djibouti (*Exaesiopus henoni*). *Exaesiopus glaucus* is known only from the Republic of South Africa and Namibia.

### Exaesiopus grossipes

Taxon classificationAnimaliaColeopteraHisteridae

(Marseul, 1855)

Figs 1 , 2–9 , 10–13 , 14–16 , 17–25 , 26–34

Saprinus grossipes Marseul 1855: 718, t. XX, fig. 153; Schmidt (1885): 315.
Saprinus rugicollis Schmidt 1890: 19 (*nomen nudum*, given as synonym).
Pachylopus grossipes : Schmidt (1896): 296; G. Müller (1931): 102.
Hypocaccus grossipes : Ganglbauer (1899): 393.
Styphrus grossipes : Jakobson (1911): 651.
Exaesiopus grossipes Reichardt (1926): 16; Reichardt (1941): 329, 330, fig. 117; Peyerimhoff (1936): 227; Kryzhanovskij and Reichardt (1976): 232, Figs 455–458; Vienna (1980): 196, fig. 69; Mazur and Kaszab (1980): 61, Figs 31, 34 D, E, F; Mazur (1984): 101; Mazur (1997): 263; Yélamos (2002): 338, Figs 12E, 161G, 169, 170A; Mazur (2004): 92; Lackner (2010): 112, Figs 19, 54, 89, 118, 339–359; Mazur (2011): 210.
Exaesiopus grossipes berberus Peyerimhoff 1936: 227 – **syn. n.**

#### Type locality.

Spain, France: Bayeux, Marseille.

#### Type material examined.

*Saprinus grossipes*: Lectotype, present designation, sex undetermined, pinned, right mesotibia, left mesotarus, both hind legs missing, with the following labels: “153 / Saprinus / grossipes / m / Marseille / Barage ?” (round illegible label, written); followed by: “MUSEUM PARIS / COLL. / DE MARSEUL 1890” (printed); followed by: “TYPE” (red-printed label); followed by: “Saprinus grossipes / Marseul, 1855 / LECTOTYPE 2014 / des. T. Lackner” (red label, written).

*Exaesiopus grossipes berberus*: Lectotype, ♀, side-mounted on a triangular point, final two metatarsomeres on right hind leg missing, with the following labels: “Laghouat” (written); followed by: “Coll. Hénon / T Théry” (written); followed by: “Saprinus / grossipes berberus / Peyerimhoff / TYPE” (written); followed by: “Exaesiopus / grossipes berberus / Peyerimhoff, 1936 / LECTOTYPE / des. T. Lackner 2014” (red label, written) (MNHN). Paralectotypes, 2 ♂♂, both mounted on a single pin on triangular mounting points with extracted genitalia, with the following label: “Bône” (written); followed by: “Coll. Hénon / T Théry” (written); followed by: “Saprinus grossipes / berberus Peyerimh / TYPE” (written); followed by: “Exaesiopus / grossipes berberus / Peyerimhoff, 1936 / PARALECTOTYPE / des. T. Lackner 2014” (red label, written) (MNHN).

#### Note.

Peyerimhoff (1936: 227) distinguished the subspecies *Exaesiopus grossipes berberus* from the nominotypical one based on elytral punctation that should cover almost the entire elytral disc basally and laterally up to the second dorsal elytral stria. Among the three specimens that he furnished with type labels, however, only the female from Laghouat (Algeria) exhibits these characteristics; the two males from Bône [=Annaba, Algeria] have their apical half (approximately) impunctate and the punctation of their elytral discs terminates in third elytral stria. Therefore, a female from Laghouat has been selected for the Lectotype. Peyerimhoff (1936: 227) himself did not specify which specimen(s) belonged to the type series; he listed several localities with his extremely brief diagnosis of the new subspecies. Both Laghouat and Bône [=Annaba] were among the listed localities.

#### Additional material examined.

Bulgaria: 1 ♀, Asenovgrad, vi.1963, A. Olexa leg. (TLAN); 1 spec., Nessebar, 30.v.1996, O. Majzlan leg. (TLAN); 1 spec., Kazanlak, vi. 1963, A. Olexa leg. (TLAN); 1 spec., Plovdiv, Rektořík leg. (TLAN); 1 ♂, Newrokop, 19.vi.1938, leg. Hlinikowski (TLAN); 1 spec., SW Bulgaria, 2 km N Gara Pirin, 11.-12.vi. 1983, leg. Hieke (NCB). BOSNIA-HERZEGOVINA: 1 ♀, Mostar, V. Zoufal leg. (ZMHUB). MACEDONIA: 2 specs., 5 km E of Velandovo, 31.v.1992, P. Zahradník leg. (TLAN). Slovakia: 1 ♂, 1 ♀ + 1 spec., Čenkov, 24.vi. 1987, V. Kubáň lgt. (TLAN); 1 spec., ibid, but, 29.v.1993, T. Růžička leg. (TLAN); 2 specs., Kameničná-Balvany 8174c, 13.vii.2000, O. Majzlan leg. (TLAN). Serbia: 1 spec., Veliko Gradište, 2.ix.1955, Stanćić leg. (TLAN); 1 spec., Vranje, 4.vi.1968, collector unknown (TLAN). ITALY: 1 ♀, Piemont, coll. Bickhardt (MNHN); 1 ♂, Torino, coll. Fea (MNHN); 1 ♂ +1 ♀, Lagnola (?), xi. 1910, Sekera leg. (TLAN); 1 spec., PO, Fiume, Piacenza, 2.vi.1963, leg. P. Ratti (NCB); 1 spec., Veneto, Caorle, v. 1999, Clereau leg. (CAS); 2 ♂♂ + 1 ♀, Ponferrada, Paganetti (ZMHUB). FRANCE: 1 ♂, Bouches-du-Rhône, Les Saintes-Maries-de-la-Mer, 18.iv.1978, P. Queney leg. (CYG); 1 ♀, Charente Maritime, île de Ré, viii.1978 (P. Queney leg. (CYG); 1 ♂+1 ♀, Gard, Le Grau-du-Roi, 31.viii.1947, J. Thérond leg. (CYG); 4 ♂♂+1 ♀, Gironde, Soulac, 12.iv.1890, E. Giraud leg. (CYG); 1 ♀, idem, but 6.vii.1975, G. Tempère leg. (CYG); 1 ♀, Manche, Portbail, beach, 22.vi.1955, H. Chevin leg. (CYG); 1 ♂, Pyrénées, (CYG); 1 ♂, Montelimar, 7.v.1912, Laboissere leg. (MNHN); 1 ♀, Plouharnel, 1878, no further data (MNHN); 5 specs., Bretagne, Nicolas, no further data (MNHN); 1 ex., Var, Nice, flooding, v. 1951 (MNHN); 3 specs., Grande Coté, Royau, v. 1918, Chobaut & R. Lebon (MNHN); 1 spec., Aveyron, Millau, 6.iv.1960, Fages lgt. (MNHN); 2 specs., Ile de Ré, coll. Bonnaire, no further data (MNHN); 3 specs., La Rochelle d’Orbigny, no further data (MNHN); 4 specs., Carcassonne, no further data (MNHN); 2 specs., Pluharnel, dept. de Morbihan, no further data (MNHN); 2 specs., St. Jean de Monts, P. Sirguey (MNHN); 1 spec., Agen, 30.v.1908, G. Nicolas (MNHN); 2 specs., Toulouse, Col. D. Grenier (MNHN); 1 spec., Grau du Roi, 11.iv.1955, J. Thérond leg. (MNHN); 2 specs., ibid, but 2.ii.1938, J. Thérond leg. (MNHN); 1 spec., ibid, but 10.ix.1948, J. Thérond leg. (MNHN); 1 spec., ibid, but 19.v.1970, J. Thérond leg. (MNHN); 1 spec., ibid, but 15.v.1951, J. Thérond leg. (MNHN); 1 spec., ibid, but 1.x.1949, J. Thérond leg. (MNHN); 2 specs., ibid, but 9.iii.1940, J. Thérond leg. (MNHN); 2 specs., Pont du Gard, 3.iii.1927, J. Thérond leg. (MNHN); 2 specs., ibid, but 29.v.1928, J. Thérond leg. (MNHN); 2 specs., ibid, but 22.ix.1931, J. Thérond leg. (MNHN); 1 spec., Camargue, St. Maries, 9.x.1928, L. Puel leg. (MNHN); 5 specs., Vendée, St. Jean de Monts, vi.1926, P. Sirguey leg. (NCB); 2 ♂♂ + 1 ♀, ibid, but MNHN; 1 spec., Morgat, Brittany, no further data (BMNH); 2 specs., France, no further data (BMNH); 1 spec., Erqny, Côtes du Nord, H.D. Preston leg. (BMNH); 1 spec., Provence, no further data (BMNH); 3 specs., St. Jean de Monts (Vendée), P. Sirguey leg., 1926 (BMNH); 1 spec., Manche, Utah Beach, 6.vi.[19]64; 1 spec., Beziers, no further data (BMNH) 1 spec., ibid but ZMHUB; 1 spec., Grau du Roi, 29.iii.1943, J. Thérond leg. (ZIN); 1 spec., Lyon, in Rhône, no data or collector (ZIN). HUNGARY: 1 spec., Hungary, no further data (BMNH); 1 ♂, Jarabszállás, 30.v.1971, P. Polák leg. (TLAN); 1 ♂, Dunakeszi, no further data (ZMHUB). SPAIN: 1 spec., Valencia, no further data (MNHN). IRAQ: 1 spec., Mesopotamia, Millingen, no further data (BMNH); 1 ♀, Mesopotamia, no further data (ZMHUB). Russia: 1 spec., Volgogradskaya obl., Tsimlya, 27.vii.1894, collector unknown (ZIN). Ukraine: 1 spec., Stan. Luganskaya, Lugansk okr., 17.vi.1928, collector unknown (ZIN); 2 ♀♀, Khersonskaya oblast, Alyoshki, Dneprovskij uezd, 26.v.1926, D. Znojko leg.; 2 specs., ibid, but 19.v.1929, N. Kostenko leg.; 1 spec., Khersonskaya oblast, Burkutskie plavni [zapovednik], 17.v.1929, N. Kostenko leg.; 1 spec., ditto, but Kazach village, 7.vi.1928, N. Kostenko leg. (all exs. ZIN). Greece: 1 spec., Peloponese occid., Epitalion, Alfios River, nr. Pyrgos, 13.iv.1995, T. Kopecký leg. (TLAN); 1 ♀, Pirgos, 1.v.1971, leg. Wewalka leg. (TLAN); 1 spec., Peloponesus, Xylokatron, 22.v.1962, H. Pochon leg. (MNHN); 1 spec., Thessalia, no further data (ZMHUB). MOROCCO: 1 ♀, Tauorirt, 10.iii.1993, G. Chavanon leg. (CYG); 1 ♂, Morocco centr., Moyen Atlas, Aguelmame Azegza lake, 22.–26.vi.1998, T. Lackner leg. (TLAN); 1 spec., Ouarzazate prov., Oued Draa River valley, Agdz env., N 30.40.52 W 006.25.08, 29.iii.2011, in human faeces, A. Gusakov leg. (CAS); 1 ♂, Beni Ounif near Figuig, 11.v.1944, Barbier leg. (MNHN). TUNISIA: 1 ♂, Medjez el Bab, v.1935, R. Demoflys leg. (CYG); 1 ♂, Gabès, v. 1944, R. Demoflys leg. (CYG); 1 spec., Zarzia, 5.–11.v.1977, M.A. Hielkema leg. (NCB). 1 ♀, Tunis, i–ii.1882, G. & L. Doria leg. (ZMHUB); 1 spec., Hammamet mer., 25.iii.-4.iv.1992, A. Pütz leg.; 2 ♀♀, 1 ♂ & 1 spec., 6-11.vi.1982, Kairuan, A. Olexa leg. (TLAN). ALGERIA: 1 ♀, Bona [=Bône?], Desbr., no further data (ZMHUB); 2 ♂ + 1 ♀, Oued Sebaou near Tizi-Ouzou, 25.vi.1908, collector unknown (MNHN); 2 ♀♀, Aïn Sefra, v. 1936, collector unknown (MNHN); 1 ♂, ibid, but coll. Bonnaire (MNHN); 1 ♀, Bou-Ktoub, S of Oran, Déchoguat leg. (MNHN); 1 ♀, Biskra, v. 1885, L. Bleuse leg. (MNHN); 1 ♀, Bou-Saada, no further data (MNHN); 1 ♀, south of Oran, no further data (MNHN) 1 ♂ + 1 ♀, Colomb-Béchar, 1912, P. Germain leg. (MNHN).

#### Redescription.

Although this species has been recently re-described by the author (Lackner 2010: 112–116), I prefer to repeat this re-description here for the reason that the following species are morphologically rather similar to *Exaesiopus grossipes*. Those species are consequently provided only with diagnostic descriptions outlining their respective differences.

Body length: PEL: 2.10–2.75 mm; APW: 0.825–1.00 mm; PPW: 1.625–2.25 mm; EL: 1.25–2.00 mm; EW: 1.875–2.50 mm.

**Figure 1.**
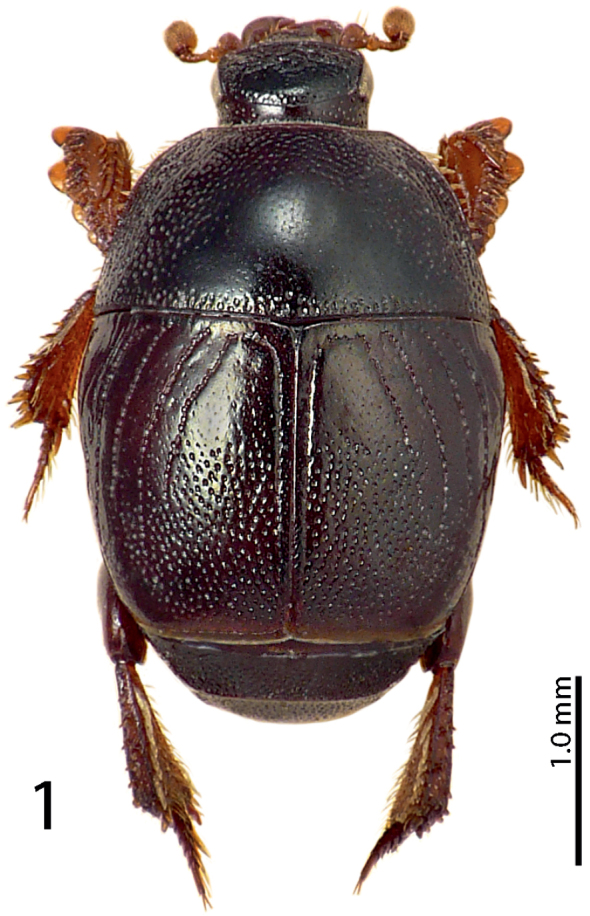
*Exaesiopus grossipes* (Marseul, 1855) habitus.

Body (Fig. 1) oval, convex, cuticle light to dark brown, sometimes with feeble bronze or greenish metallic tinge; legs, mouthparts and antennae rufopiceous.

**Figures 2–9.**
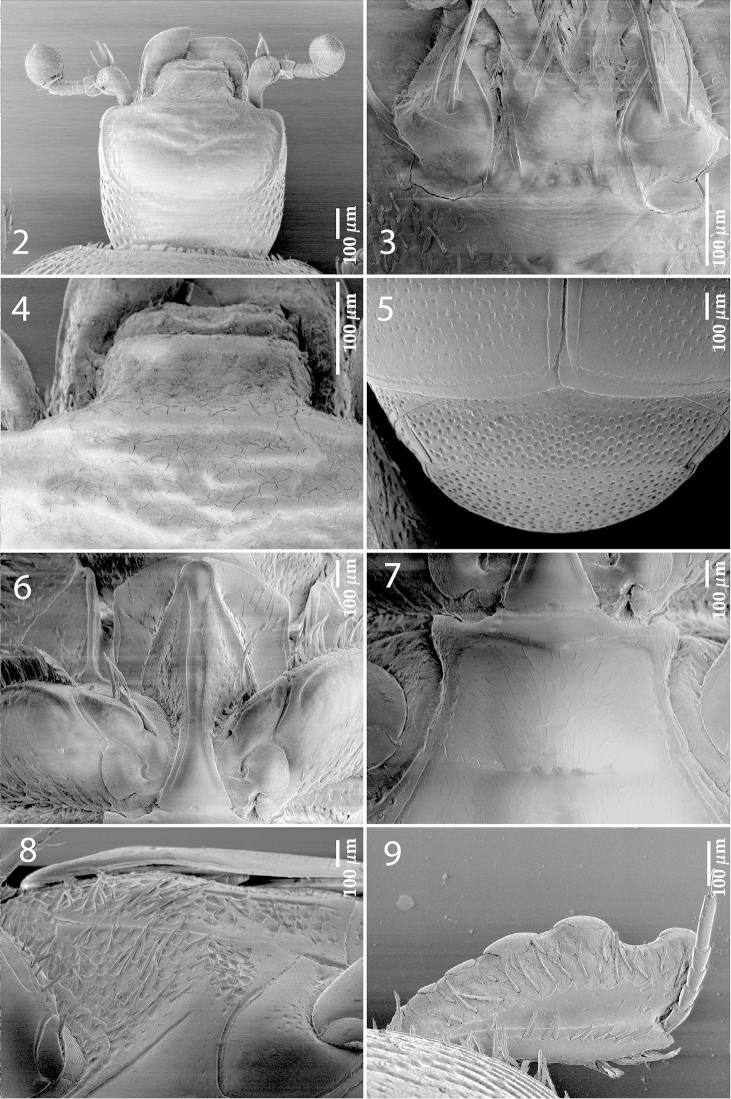
*Exaesiopus grossipes* (Marseul, 1855) **2** head, dorsal view **3** mentum, ventral view **4** clypeus **5** propygidium + pygidium **6** prosternum **7** mesoventrite **8** lateral disc od metaventrite + metepisternum **9** protibia, dorsal view.

Antennal scape with few short setae; club (Fig. 2) round, entire surface with thick short yellow sensilla intermingled with sparse slightly longer setae; sensory structures of the antennal club (Fig. 14) in form of stipe-shaped vesicle situated under circular sensory area on internal distal margin of the ventral side of antennal club.

Mouthparts: mandibles (Fig. 15) stout, outer margin slightly curved; mandibular apex bluntly pointed; sub-apical tooth of left mandible large, almost perpendicular; labrum (Fig. 16) sparsely punctate, shallowly depressed medially, two labral pits present, two labral setae arising from each; epipharynx almost completely hidden under labral fold; terminal labial palpomere elongated, its width less than half its length; mentum (Fig. 3) square-shaped, with deep antero-median notch; anterior margin with few long setae, lateral margins with single row of sparse shorter ramose setae; cardo of maxilla with few short setae on lateral margin; stipes triangular, with three much longer setae; terminal maxillary palpomere somewhat thickened, its width less than half its length, about twice as long as penultimate.

Clypeus (Fig. 4) rectangular, almost smooth, can be slightly rugose, anterior margin elevated, clypeus depressed medially; frontal stria well impressed, almost straight (sometimes somewhat curved outwardly), carinate, continued as carinate supraorbital and postorbital striae; frons with two to several irregularly shaped carinate transverse rugae or chevrons; eyes flattened, inconspicuous from above.

Pronotal sides slightly convergent forwards; apical angles blunt; marginal stria complete; pronotal disc convex, with round dense punctation, forming transverse rugae laterally, postero-median part of disc usually smooth, at times entire disc punctate (punctation can also stop short of lateral pronotal margin); pronotal base with a double row of round dense punctures; pronotal hypomeron with amber setae; scutellum small, visible.

Elytral humeri slightly prominent, elytra broad, almost as broad as long at its widest point; elytral epipleura with microscopic punctures, almost smooth; marginal epipleural stria complete; marginal elytral stria deeply impressed, continued as well impressed apical elytral stria; regular row of round punctures present along elytral marginal stria. Humeral elytral stria weakly impressed on basal third, sometimes doubled; inner subhumeral stria present medially, deep and rather long, rarely joining marginal elytral stria; elytra with four dorsal punctate elytral striae 1–4, all striae approximately reaching elytral half apically (occasionally slightly surpassing it), fourth elytral stria basally connected with sutural elytral stria; sutural stria deeply punctured, apically joining apical elytral stria. Elytral punctation variable, often confined to apical half of elytra, along elytral suture reaching almost anterior third of elytral disc, punctures regular and deep, separated by about half to their own diameter, occasionally (often in specimens from North Africa) covering most part of elytral disc (elytral flanks and humeri almost always smooth).

Propygidium (Fig. 5) almost completely exposed, long, covered with coarse and dense regular punctation; punctation of pygidium (Fig. 5) sparser and finer, punctures separated by about 1–3 times their diameter.

Anterior margin of median portion of prosternum (Fig. 6) regularly rounded; prosternal foveae weakly to well impressed, small and often indiscernible under conventional binocular microscope; prosternal process slightly to deeply concave, dorsally impunctate, laterally substrigulate-punctate, few microscopic setae present; carinal prosternal striae divergent between procoxae, subparallel, vaguely united in front, at times obliterated on their anterior third; lateral prosternal striae well impressed, carinate, convergent anteriorly, united in front of apices of carinal prosternal striae.

Mesoventral disc (Fig. 7) somewhat convex, almost smooth, slightly wider than long; meso-metaventral sutural stria well impressed, with several accompanying punctures; intercoxal disc of metaventrite with longitudinal depression in male, smooth, basally with irregular sparse shallow fine punctures; lateral metaventral stria (Fig. 8) well impressed, carinate, obliquely arcuate, apically almost reaching metacoxa; lateral disc of metaventrite concave, with shallow setiferous punctures of various sizes, separated by approximately their own diameter; metepisternum with even denser and coarser punctation and setae, on apical third + metepimeron punctation much finer and sparser; metepisternal stria deeply impressed, present on metepimeron and approximately apical third of metepisternum.

Intercoxal disc of first abdominal sternite almost completely striate laterally; disc almost smooth, with sparse punctures along apical margin; lateral portion of disc of all visible abdominal sternites with short setae.

**Figures 10–13.**
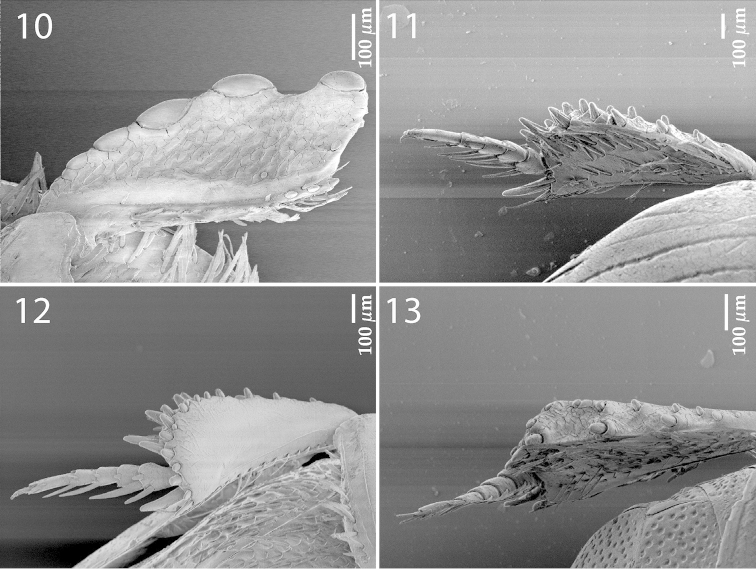
*Exaesiopus grossipes* (Marseul, 1855) **10** protibia, ventral view **11** mesotibia, dorsal view **12** ditto, ventral view **13** metatibia, dorsal view.

Protibia (Fig. 9) on outer margin with two to three low teeth, topped with triangular to rounded (blunt, if worn) denticle followed by two inconspicuous rounded denticles; setae of outer row sparse, moderately long; setae of median row shorter than those of outer row, sparse; anterior protibial stria shortened apically; protibial groove shallow; protibial spur (Fig. 10) minuscule, growing out from apical margin of protibia; outer part of posterior surface of protibia (Fig. 10) obscurely variolate, vaguely separated from comparatively narrower median part, posterior protibial stria complete, terminating in two minute inner posterior denticles; inner margin of protibia with double row of short dense ramose setae.

Mesotibia (Fig. 11) moderately dilated and thickened, outer margin with two rows of sparse short denticles; setae of outer row well sclerotized, comparatively short; setae of median row shorter and sparser, covering most of posterior surface; posterior mesotibial stria vaguely impressed, shortened apically; mesotibial spur stout, prominent and long; anterior face of mesotibia (Fig. 12) smooth; anterior mesotibial stria shortened apically; claws of last tarsomere bent, shortened, shorter than half its length.

**Figure 14–16.**
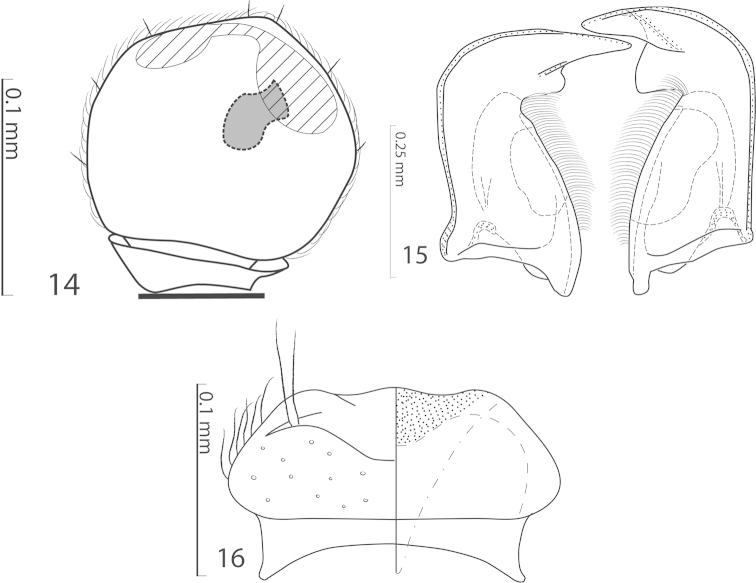
**14** *Exaesiopus grossipes* (Marseul, 1855) sensory structures of the antenna **15** mandibles, dorsal view **16** labrum, left half depicting dorsum; right half depicting underside.

Metatibia (Fig. 13) triangularly dilated and thickened apically; outer margin with four widely-spaced short rounded denticles, a single row of tiny sparse rounded denticles present dorsally on thickened anterior face of metatibia; setae of intermedian row shorter and denser, cover almost the entire posterior face of metatibia; otherwise metatibia similar to mesotibia.

**Figures 17–25.**
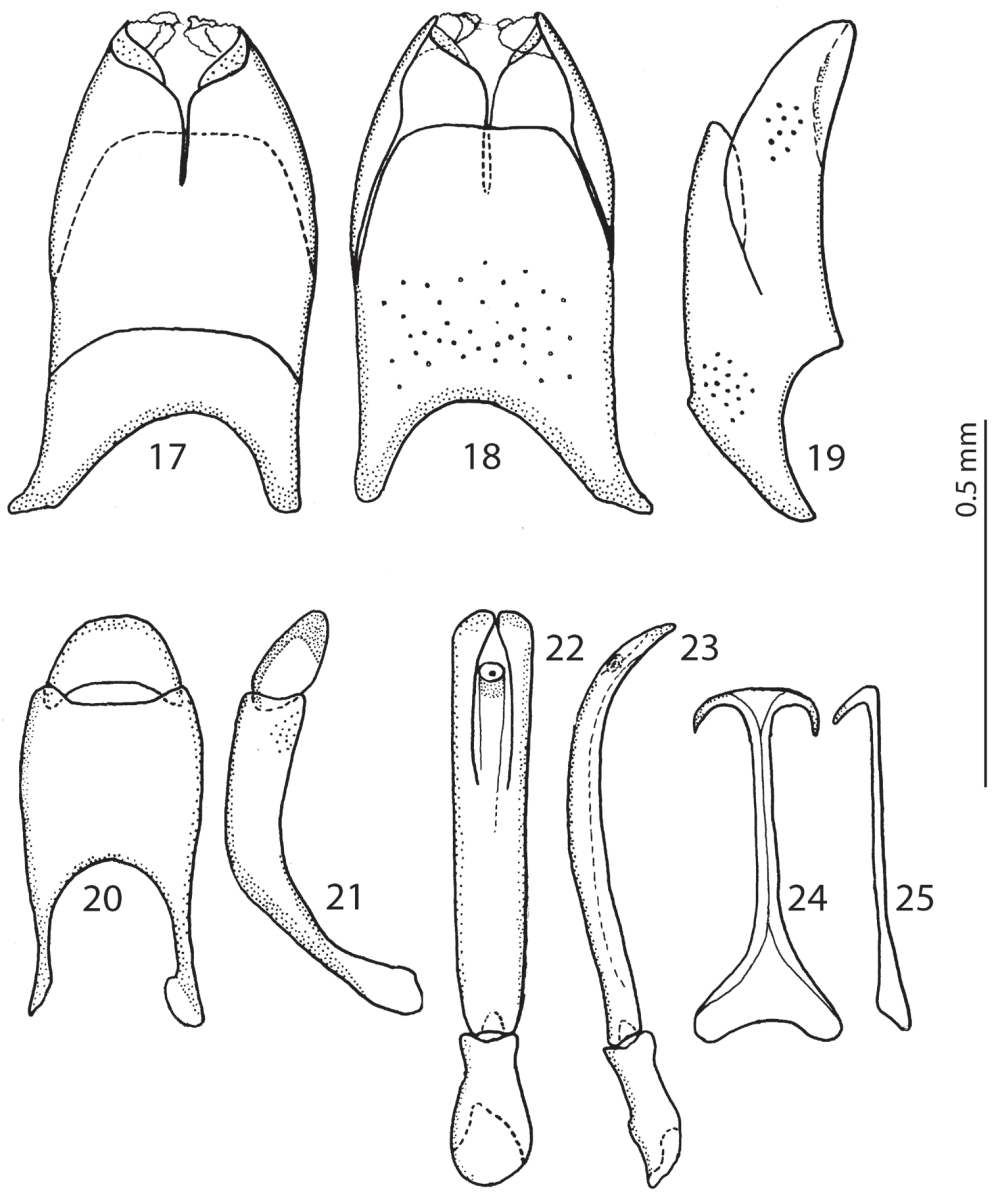
*Exaesiopus grossipes* (Marseul, 1855) from Bulgaria, 8^th^ sternite and tergite, **17** ventral view **18** ditto, dorsal view **19** ditto, lateral view **20** 9^th^ + 10^th^ tergites, dorsal view **21** ditto, lateral view **22** aedeagus, dorsal view **23** ditto, lateral view **24** spiculum gastrale, ventral view **25** ditto, lateral view.

Male genitalia. Eighth sternite (Figs 17–18) on apical half longitudinally separated medially, with tiny asetose vela, eighth sternite and tergite fused laterally (Fig. 19). Ninth tergite (Figs 20–21) apically with faint emargination; basally deeply emarginated; tenth tergite (Fig. 20) apically outwardly arcuate, basally faintly inwardly arcuate. Spiculum gastrale (Figs 24–25) typical for the subfamily representing the most common type with ‘head’ and ‘tail’ (sensu Caterino & Tishechkin, 2013); ‘tail’ cordate, ‘head’ with two narrow, curved arms. Aedeagus (Figs 22–23) tube-like, slender, basal piece of aedeagus rather short, ratio of its length: length of parameres 1:3.5; parameres fused along their basal two-thirds, aedeagus slightly curved ventrad (Fig. 23).

#### Differential diagnosis.

**Figures 26–34.**
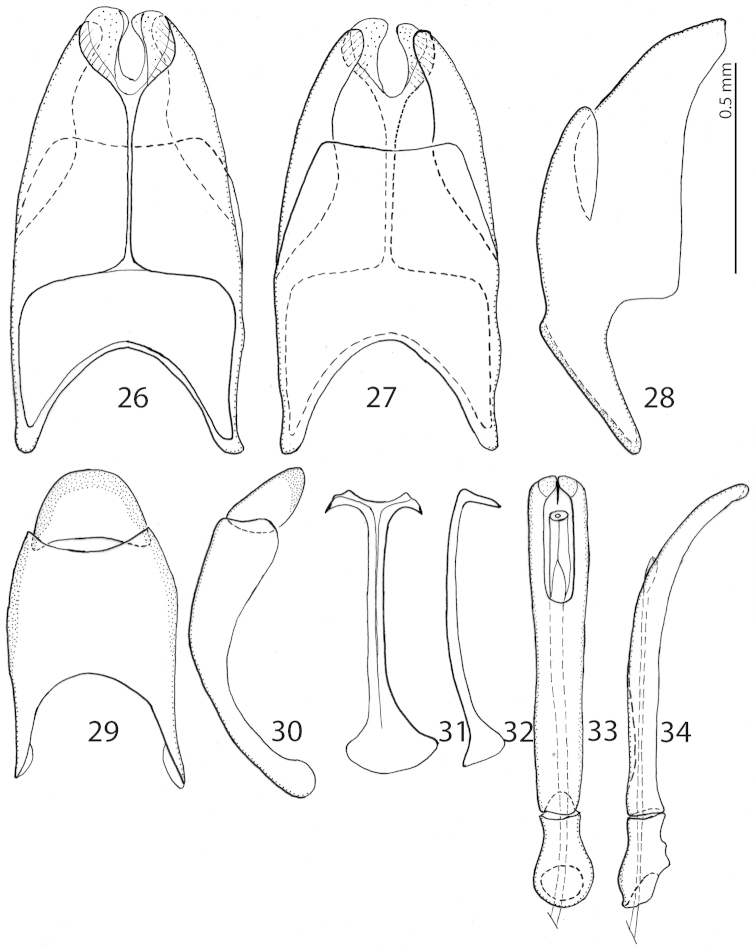
*Exaesiopus grossipes* (Marseul, 1855) from Tunisia, 8^th^ sternite and tergite, **26** ventral view **27** ditto, dorsal view **28** ditto, lateral view **29** 9^th^ + 10^th^ tergites, dorsal view **30** ditto, lateral view **31** spiculum gastrale, ventral view **32** ditto, lateral view **33** aedeagus, dorsal view **34** ditto, lateral view.

*Exaesiopus grossipes* differs from the three species *Exaesiopus henoni*, *Exaesiopus therondi* and *Exaesiopus laevis* chiefly by the shape of its protibia, which is on its outer margin furnished with three low teeth topped by triangular or rounded denticles (Figs 9, 10), whereas the three other mentioned species have their protibia furnished with two large teeth topped by triangular denticles on outer margin (Figs 61, 79 & 114). From *Exaesiopus atrovirens* and *Exaesiopus glaucus* it differs chiefly by the absence of a green metallic hue of the dorsum (compare Figs 1 with 73 & 98); from *Exaesiopus glaucus* it differs furthermore by thickened and dilated metatibia (compare Figs 13 with 106). On the other hand, some specimens of *Exaesiopus grossipes* (especially from N. Africa that formerly belonged to the subspecies *berberus*) can resemble the specimens of Middle-Asian *Exaesiopus torvus* by their densely punctate dorsum. These specimens differ, however, from *Exaesiopus torvus* by their respective male genitalia (compare Figs 17–34 with 64–72) and the less punctate pronotal disc (see also Key to the species for details, below). Most specimens of *Exaesiopus grossipes*, however (especially those from the northern shore of the Mediterranean Sea and South Europe) have distinctly less punctate dorsum than the specimens of *Exaesiopus torvus*.

#### Biology.

This species is found on the beach under coastal wrack as well as further away from the waterfront, almost exclusively on sandy soil. Beetles can be found under rotting fish, excrements or buried under vegetation.

#### Distribution.

Known from the Canary Islands, Morocco, Algeria, Tunisia, Libya, Spain, France, Italy, Greece, Bosnia and Herzegovina, Macedonia, Bulgaria, Russia, Serbia, Slovenia, Ukraine, Slovakia, Hungary, Austria, Iraq.

#### Remarks.

A variable species, covering vast area from the Canary Islands in the west to Iraq in the east. Its external morphology as well as male genitalia exhibit a certain degree of variation (compare Figs 17–25 and 26–34), but I find it difficult to discern discrete states among the variation and prefer to lump all examined specimens under the same species.

### Exaesiopus henoni

Taxon classificationAnimaliaColeopteraHisteridae

(Schmidt, 1896)

Figs 35 , 36–43 , 44–45 , 46–54

Pachylopus henoni Schmidt 1896: 206
Saprinus henoni : Bickhardt 1910: 106.
Exaesiopus henoni : Mazur (1984): 101; (1997): 264; (2004): 92; (2011): 210.

#### Type locality.

Aïn Sefra, Algeria.

#### Type material examined.

Lectotype, ♀, side-mounted on triangular point, left metatarsus missing, with the following labels: “♀” (printed); followed by: “henoni m / Aïn Sefra” (written); followed by: “coll Schmidt- / Bickhardt” (printed); followed by: “Pachylopus / henoni Schmidt / Coll. Schmidt-Bickhardt” (printed); followed by: “LECTOTYPE / N. Dégallier” (red label, printed) (ZMHUB). 1 ♂ paralectotype, with the following labels: “Aïn Sefra / Hénon” (printed); followed by: “Pachylopus / Henoni / Schm. Type” (written); followed by: “PARA- / LECTOTYPE / N. Dégallier” (printed) (BMNH). 1 ♀, paralectotype, with the following labels: “Aïn Sefra / Hénon” (printed); followed by: “Pachylopus / Henoni / Cotype ‘96 Sch.” (written); followed by: “PARA- / LECTOTYPE / N. Dégallier” (printed) (BMNH); Paralectotypes: 1 ♂ & 4 specs., with the following labels: “Aïn-Sefra / Hénon” (printed); followed by: “Museum Paris / ex coll. / P. de Peyerimhoff” (printed); followed by: “PARA - / LECTOTYPE / N. Dégallier” (red label, printed) (MNHN).

1 Syntype, ♀, side-mounted on a triangular point, with the following labels: “♀” (printed); followed by: “Type” (brick-red label, printed); followed by: “Aïn-Sefra / Hénon” (printed); followed by: “Pachylopus / Henoni typ” (written); followed by: “Pachylopus / henoni Schmidt / Coll. Schmidt-Bickhardt” (printed); 1 Syntype, ♀, side-mounted on a triangular point, with the following labels: “♀” (printed); followed by: “Type” (brick-red label, printed); followed by: “Aïn-Sefra / Hénon” (printed); followed by: “Pachylopus / Henoni m” (written); followed by: “coll. J. Schmidt” (printed); followed by: “Pachylopus / henoni Schmidt / Coll. Schmidt-Bickhardt” (printed); 1 Syntype, ♀, side-mounted on a triangular point, with the following labels: “♀” (printed); followed by: “Type” (brick-red label, printed); followed by: “Aïn-Sefra / Hénon” (printed); followed by: “Pachylopus / Henoni m” (written); followed by: “Pachylopus / henoni Schmidt / Coll. Schmidt-Bickhardt” (printed) (all syntypes ZMHUB).

#### Additional material examined.

ALGERIA: 1 spec., Mraier, D. de Constantine, coll. de Vauloger (ZIN); 4 ♂♂ + 1 ♀ + 3 specs., Aïn Sefra, Hénon (MNHN); 1 spec., idem, but, v-vi.1896, L. Bleuse leg. (MNHN); 1 ♂ + ♀, idem, but CYG; 3 specs., Colomb-Béchar, 27.iv.1923, J. Thérond leg. (MNHN); 1 spec., ibid, but 30.iv.1923 (MNHN); 2 specs., Mraier, D. de Constantine, Vauloger (MNHN); 1 spec., Biskra, Dr. H.J. Veth leg. (NCB); 3 specs., Aïn Sefra, 26.iv.1987, A. Olexa (TLAN); 1 ♀, ibid, but 25.–27.iv.1987, D. Král leg. Djibouti: 1 ♀, As-Eyla, viii.1976 (NCB). Libya: 1 spec., Tripolitania, Wadi Sofeggin, 21.–23.v.1963, no collector (MNHN).

#### Diagnostic description.

**Figure 35.**
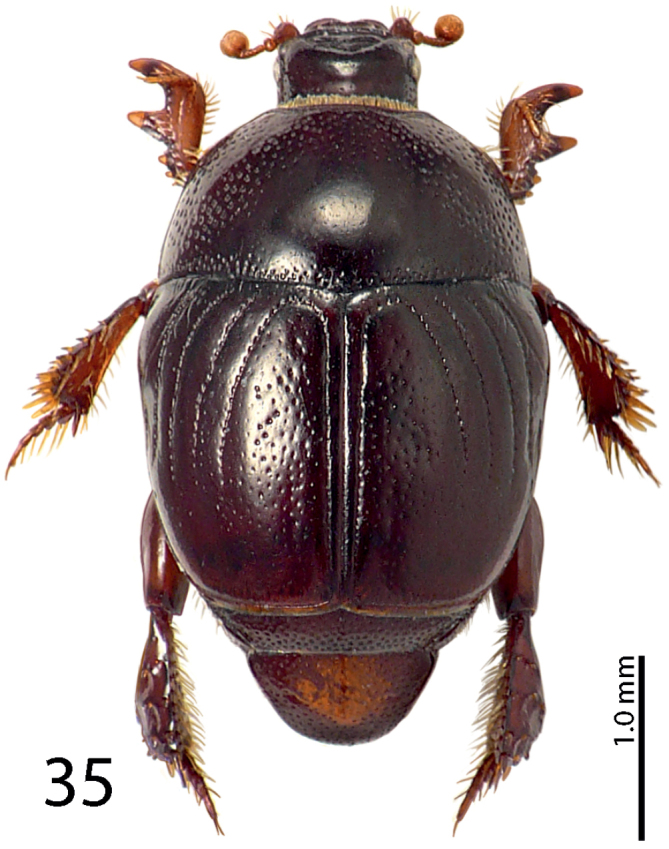
*Exaesiopus henoni* (Schmidt, 1896) habitus.

**Figures 36–43.**
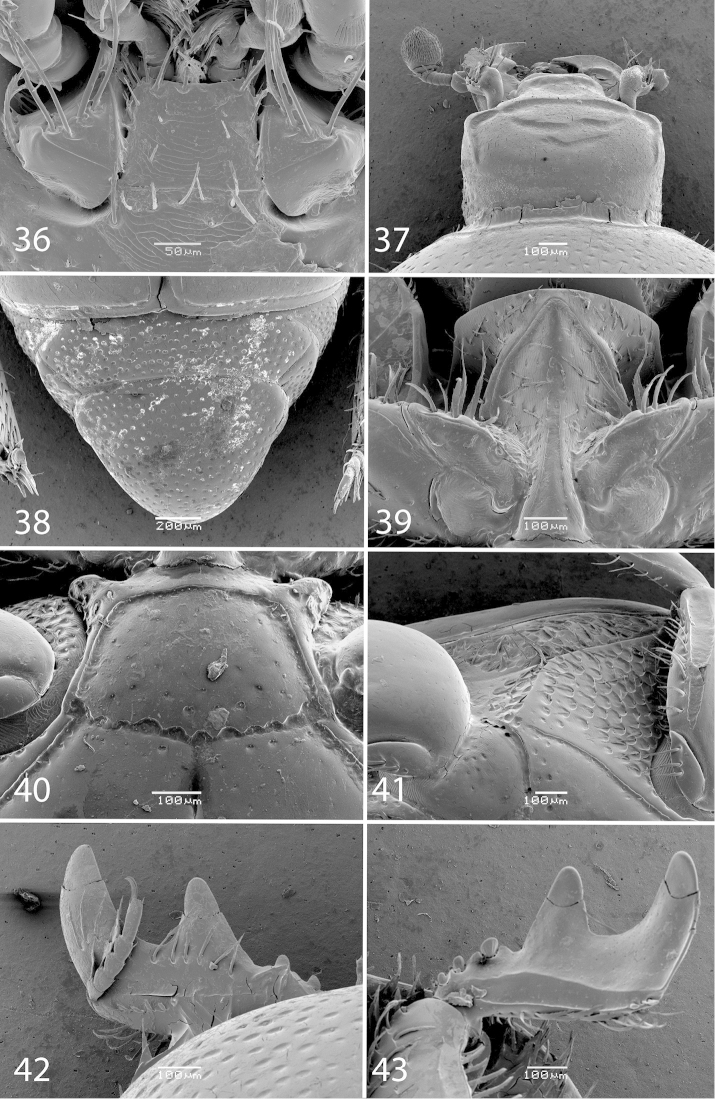
*Exaesiopus henoni* (Schmidt, 1896) **36** mentum, ventral view **37** head, dorsal view **38** propygidium and pygidium **39** prosternum **40** mesoventrite **41** lateral disc of metaventrite + metepisternum **42** protibia, dorsal view **43** ditto, ventral view.

Body length: PEL: 2.50–2.75 mm; APW: 0.875–1.00 mm; PPW: 1.875–2.00 mm; EW: 2.125–2.20 mm; EL: 1.625–1.80 mm. Body (Fig. 35) similar to *Exaesiopus grossipes*, but without any trace of metallic tinge; antennae similar to those of *Exaesiopus grossipes*; sensory structures of the antennal club not examined. Mouthparts generally similar to those of *Exaesiopus grossipes*; mentum (Fig. 36) sub-quadrate, feebly inwardly arcuate on anterior margin; anterior margin with several long setae, lateral margins with single row of sparse shorter ramose setae; stipes of maxilla with four setae (three in *Exaesiopus grossipes*); rest of the mouthparts as in *Exaesiopus grossipes*. Clypeus (Fig. 37) as in *Exaesiopus grossipes*, almost smooth; frontal and supraorbital striae as in *Exaesiopus grossipes*; postorbital stria missing (present in *Exaesiopus grossipes*); frons with two deep chevrons.

**Figures 44–45.**
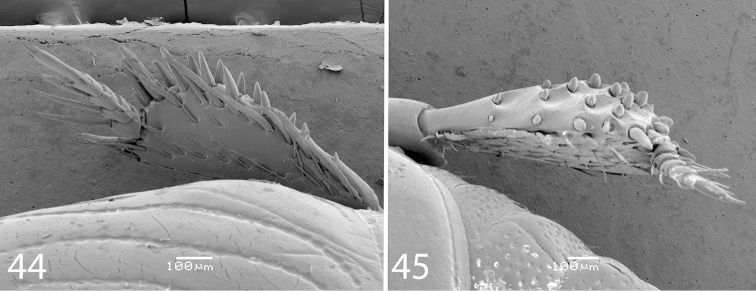
*Exaesiopus henoni* (Schmidt, 1896) **44** mesotibia, dorsal view **45** metatibia, dorsal view.

Pronotal disc (Fig. 35) with ellipsoid to round, rather sparse punctation, punctures separated by their own to several times their diameters, postero-median part of disc always smooth, punctation stopping short of lateral pronotal margin leaving a narrow impunctate band; rest of the pronotum as in *Exaesiopus grossipes*. Elytra generally as in *Exaesiopus grossipes*; inner subhumeral stria shortly present medially; dorsal elytral striae for short distance surpassing elytral half; elytral punctation variable, in most specimens present only on fourth elytral interval, but can also at times be present on other elytral intervals (a specimen from Libya), or almost completely missing (a specimen from Algeria); along elytral suture can reach almost elytral base, punctures irregular, variously deep, separated often by several times their own diameter, elytral flanks and humeri always smooth. Propygidium (Fig. 38) and pygidium similar to those of *Exaesiopus grossipes*; punctation somewhat sparser (compare Figs 5 and 38). Prosternum (Fig. 39) generally similar to that of *Exaesiopus grossipes*, but prosternal foveae very weakly impressed, often indiscernible (absent?); prosternal process deeply concave, constricted, prosternal structures and configuration of the two sets of prosternal striae similar to those of *Exaesiopus grossipes*. Disc of mesoventrite (Fig. 40) almost smooth, similar to that of *Exaesiopus grossipes*, but almost as long as wide; meso-metaventral sutural stria undulate; intercoxal disc of metaventrite with longitudinal depression in both sexes, more prominent in male, smooth, basally with several irregular rows of sparse punctures; lateral metaventral stria (Fig. 41) obliquely arcuate, apically almost reaching metacoxa; lateral disc of metaventrite (Fig. 41) and metepisternum generally similar to those of *Exaesiopus grossipes*, but metepisternum with denser and coarser punctation and longer setae; meterpisternal stria unrecognizable beneath setae (absent?). Intercoxal disc of first abdominal sternite as in *Exaesiopus grossipes*. Protibia (Figs 42–43) on outer margin with a single massive triangular tooth, followed by another lower tooth; both teeth topped by triangular denticle followed by two-three inconspicuous rounded denticles entombed in outer protibial margin; protibial spur inconspicuous (absent?); outer part of posterior surface of protibia (Fig. 43) smooth, separated from comparatively narrower median part by a definite ridge, posterior protibial stria complete, terminating in two minuscule inner posterior denticles; inner margin of protibia with double row of long dense lamellate setae. Mesotibia (Fig. 44) as in *Exaesiopus grossipes*, but denticles on outer margin longer. Metatibia (Fig. 45) even more triangularly dilated and thickened than that of *Exaesiopus grossipes*; outer margin with about four strong denticles larger in size apically; dilated anterior margin dorsally with several irregular rows of scattered tiny rounded denticles.

**Figures 46–54.**
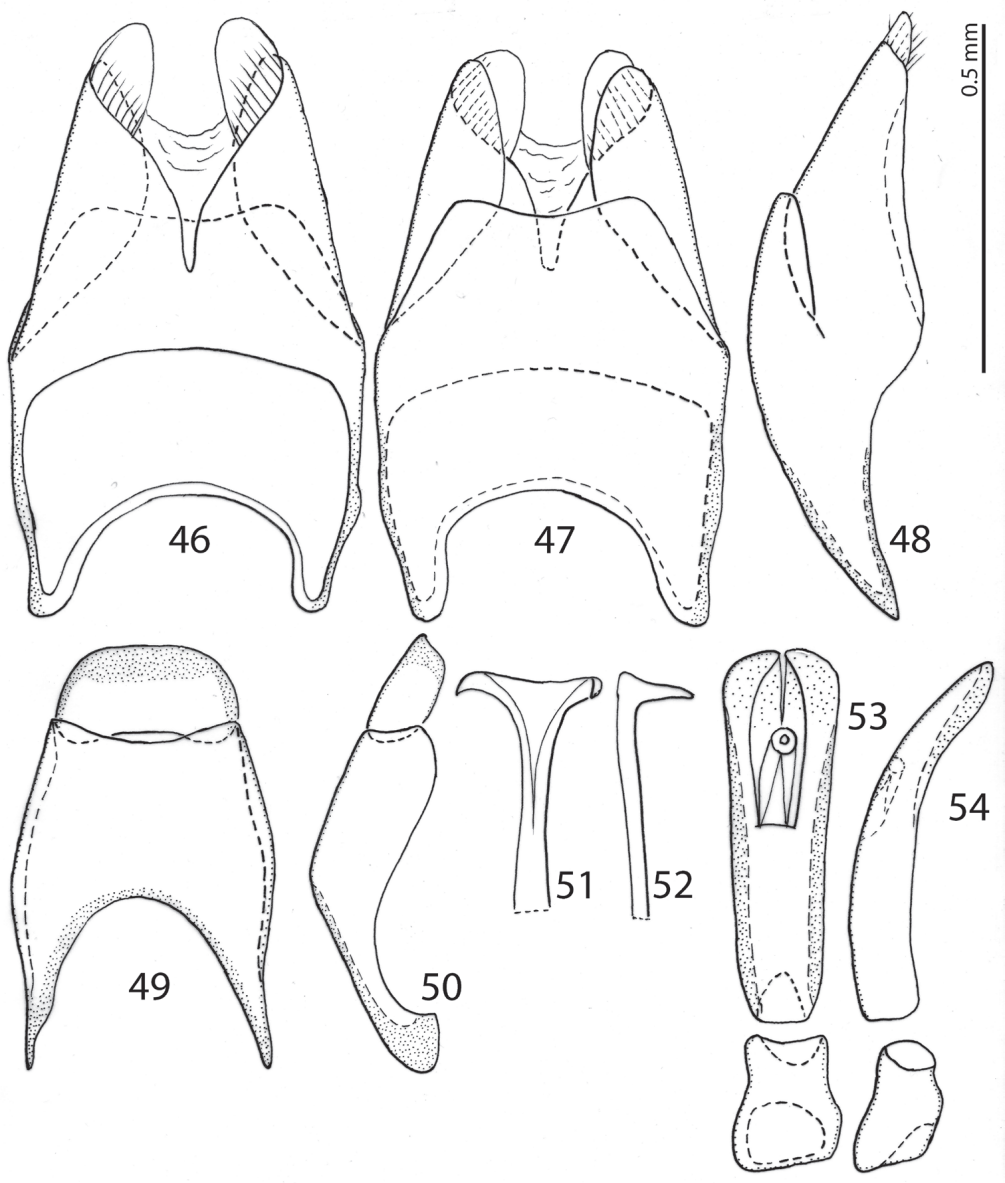
*Exaesiopus henoni* (Schmidt, 1896) 8^th^ sternite and tergite, **46** ventral view **47** ditto, dorsal view **48** ditto, lateral view **49** 9^th^ + 10^th^ tergite, dorsal view **50** ditto, lateral view **51** spiculum gastrale, ventral view **52** ditto, lateral view **53** aedeagus, dorsal view **54** ditto, lateral view.

Male genitalia. Eighth sternite (Figs 46–47) apically with a brush of sparse setae and a moderately sized velum; eighth tergite apically faintly inwardly arcuate; eighth sternite and tergite fused laterally (Fig. 48). Ninth tergite (Fig. 49) apically faintly inwardly arcuate; tenth tergite apically rounded; spiculum gastrale (Figs 51–52) similar to that of *Exaesiopus grossipes*. Aedeagus (Figs 53–54) short and stout, slightly dilated apically; apex with pseudopores; parameres fused on their apical half. Basal piece of aedeagus short; ratio basal piece: parameres approximately 1:4.

#### Differential diagnosis.

*Exaesiopus henoni* is most similar to the species *Exaesiopus laevis* and *Exaesiopus therondi*, with which it shares the shape of protibia (see also Key to species for details). From *Exaesiopus therondi* it differs by sparsely punctate pronotum, frons that is devoid of tiny irregular rugae, and anterior face of protibia, which is glabrous in *Exaesiopus henoni*, whereas it is obscurely variolate in *Exaesiopus therondi*. From *Exaesiopus laevis* it differs by punctate body (almost impunctate in *Exaesiopus laevis*) and present inner subhumeral stria (absent from *Exaesiopus laevis*). From the remaining species of the genus *Exaesiopus henoni* differs by the shape of the protibia (see also Key to species for details).

#### Biology.

A typical psammophile, found in sand.

#### Distribution.

So far known only from Algeria and Morocco (Gomy et al. 2011). New to Libya and Djibouti.

### Exaesiopus torvus

Taxon classificationAnimaliaColeopteraHisteridae

Reichardt, 1926

Figs 55 , 56–63 , 64–72

Exaesiopus torvus Reichardt 1926: 17; Mazur (1984): 101; (1997): 264; (2004): 92; (2011): 210.

#### Type locality.

Yanidarya, Kyzyl-Ordinskij Rayon, Kazakhstan.

#### Type material examined.

Holotype, ♀ side-mounted on a triangular point, with female genitalia extracted and glued to the subsequent label with female sign, with following labels: “♀” (printed); followed by circular golden label; followed by: “Yany - Darya / perovsk u / Kyzyl-Kum / 24.iv.[1]911” (hand-written); followed by: “Type / Exaesiopus / torvus m. / A. Reichardt det.” (written-printed label); followed by: “Holotypus” (red label, printed) (ZIN).

#### Additional material examined.

Kazakhstan: 1 ♀, left bank of the river Ural, Saraichikovsk, 8.vi.1932, Lukyanovich leg.; 1 ♀ + 2 specs., left bank of Ural river, opposite of Saraychik, 8.vi.1932, Lukyanovich leg.; 1 ♂, western bank of Aral Sea, Komsomolsk na Ustyurte, 31.v.1978, G. Medvedev; 1 spec., W Kazakhstan obl. [=reg.], Bilj-Agach, 4.vii.1952, L. Arnoldi (in leaf litter). (all exs. ZIN); 1 spec., 1933-102, left bank of the Ural River, Saraychik, 8.vi.[19]32, Lyukanovich (BMNH). Uzbekistan: 1 ♀, Karakum, Khiva, 3.v.1978, leg. Olexa; 1 ♂, ibid, but 1.–5.v.1979 (both exs. TLAN); RUSSIA: 1 spec., Astrakhan Region, Krasniy Yar district, near Dosang vill., 8.v.2009, A. Kovalyov leg. (CAS); 1 spec., ibid, but 14 km NE Dosang vill., barkhan [=sand dune] Tuvayak, 23-24.iv.2008, M. Smirnov leg. (CAS).

#### Diagnostic description.

**Figure 55.**
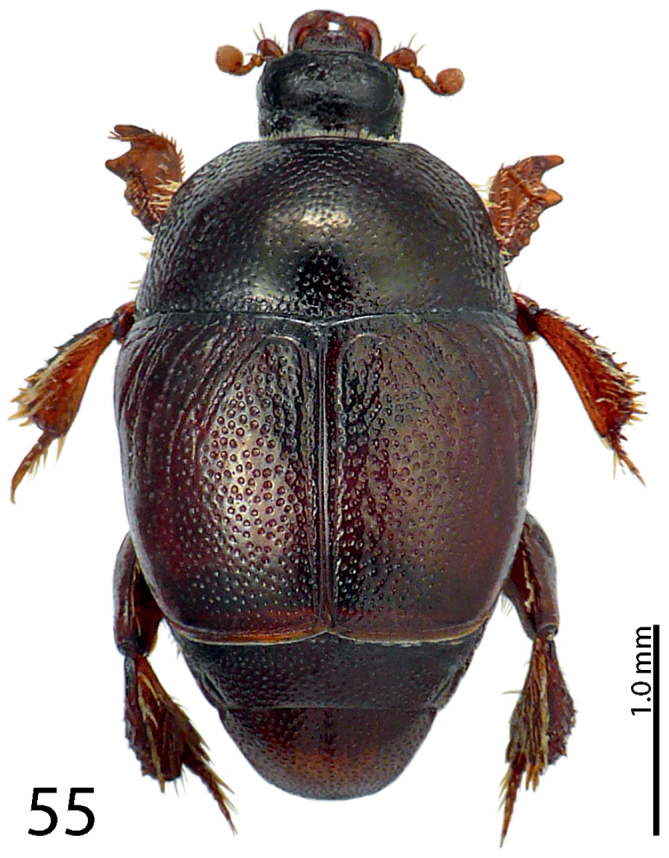
*Exaesiopus torvus* Reichardt, 1926 habitus.

**Figures 56–63.**
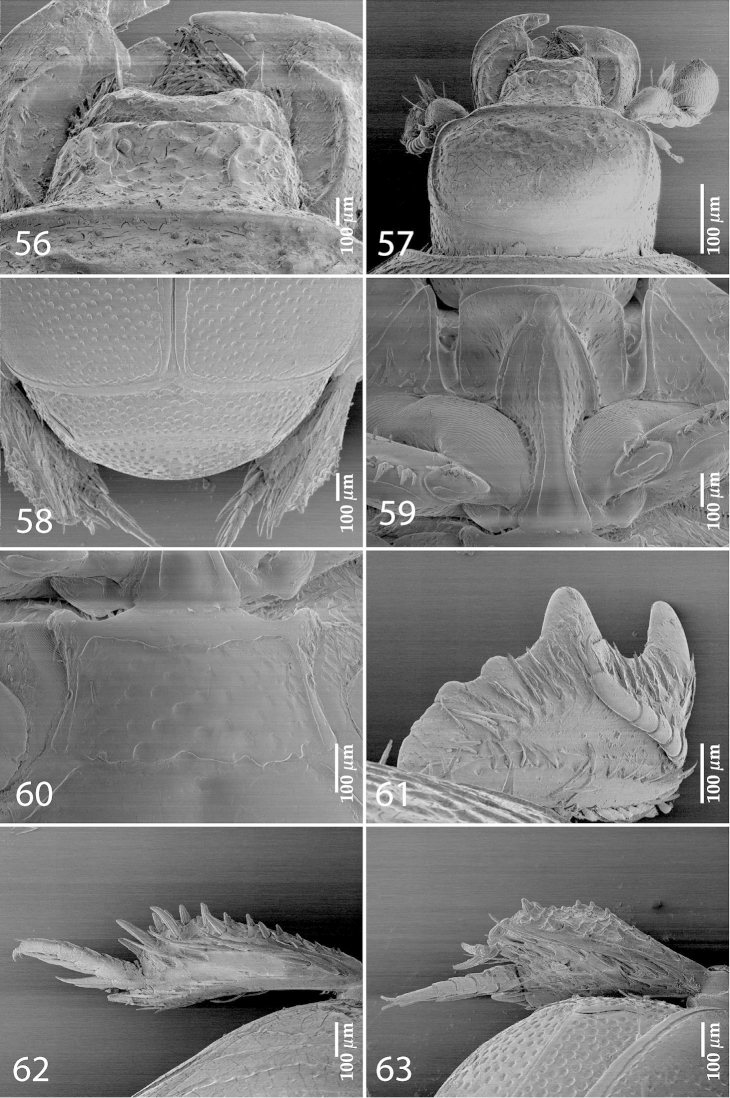
*Exaesiopus torvus* Reichardt, 1926 **56** clypeus **57** head, dorsal view **58** propygidium + pygidium **59** prosternum **60** mesoventrite **61** protibia, dorsal view **62** mesotibia, dorsal view **63** metatibia, dorsal view.

Body length: PEL: 2.15–2.575 mm; APW: 0.575–0.875 mm; PPW: 1.625–1.925 mm; EW: 1.75–2.125; EL: 1.375–1.625 mm. Body (Fig. 55) strongly convex, cuticle as in *Exaesiopus grossipes*, but without metallic tinge; legs, mouthparts and antennae dark yellow to light brown. Antennae as in *Exaesiopus grossipes*; sensory structures of the antennal club not examined. Mouthparts: labrum obscurely variolate, shallowly depressed medially; mentum as in *Exaesiopus henoni*; rest of the mouthparts similar to those of *Exaesiopus grossipes*; terminal labial and maxillary palpomeres truncate. Clypeus (Fig. 56) as in *Exaesiopus grossipes*, but rugulose-lacunose; frontal, supraorbital and postorbital striae (Fig. 57) as in *Exaesiopus grossipes*; frons rugose, with several irregularly shaped carinate transverse rugae or chevrons intermingled with sparse microscopic punctures. Pronotum: pronotal disc, except for irregularly-shaped impunctate (or weakly punctate) median part entirely covered with round dense punctation, forming transverse rugae and confluent laterally, punctation reaches lateral margins; rest of pronotum as in *Exaesiopus grossipes*.

Elytral humeri not particularly enlarged; inner subhumeral stria present only as a row of several punctures; elytral punctation variable, in most specimens reaching elytral base along fourth elytral interval, punctures often present in all elytral intervals, elytral flanks impunctate; punctures regular and deep, separated by about half to several times their own diameter. Propygidium (Fig. 58) and pygidium as in *Exaesiopus grossipes*, but covered with denser punctation. Prosternum: prosternal foveae (Fig. 59) weakly impressed; prosternal process otherwise similar to that of *Exaesiopus grossipes*. Disc of mesoventrite (Fig. 60) with scattered shallow punctures; intercoxal disc of metaventrite, lateral disc of metaventrite and metepisternum generally similar to those of *Exaesiopus henoni*. Intercoxal disc of first abdominal sternite as in *Exaesiopus grossipes*. Protibia (Fig. 61) more dilated than that of *Exaesiopus grossipes*; on outer margin with two widely-spaced low teeth, topped by large triangular denticle followed by two low rounded denticles imbedded in outer protibial margin; protibial spur inconspicuous (absent?) protibia otherwise similar to that of *Exaesiopus grossipes*. Mesotibia (Fig. 62) generally similar to that of *Exaesiopus grossipes*. Metatibia (Fig. 63) perhaps most triangularly dilated and thickened of all congeners; outer margin with approximately three widely-spaced tiny denticles; inner margin with a dense row of minuscule rounded denticles; no rows of denticles present between the two rows, surface rugulose-lacunose.

**Figures 64–72.**
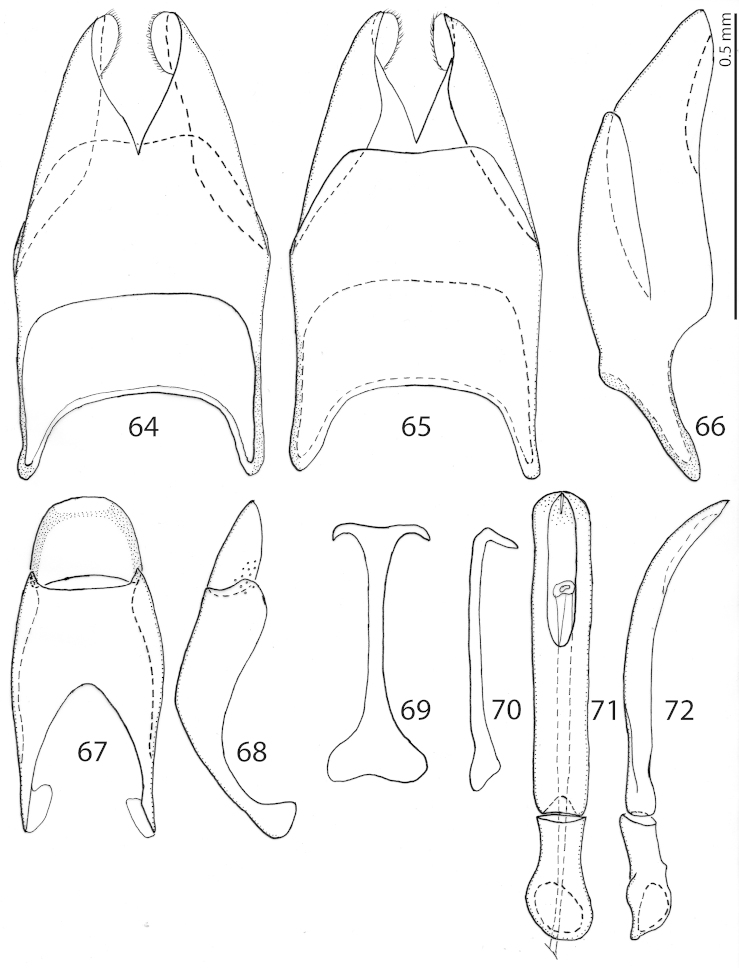
*Exaesiopus torvus* Reichardt, 1926 8^th^ sternite + tergite, **64** ventral view **65** ditto, dorsal view **66** ditto, lateral view **67** 9^th^ + 10^th^ tergites, dorsal view **68** ditto, lateral view **69** spiculum gastrale, ventral view **70** ditto, lateral view **71** aedeagus, dorsal view **72** ditto, lateral view.

Male genitalia. Eighth (Figs 64–65) sternite apically asetose, with tiny vela; eighth tergite apically faintly inwardly arcuate medially; eighth sternite and tergite fused laterally (Fig. 66). Ninth tergite (Figs 67–68) apically almost straight; tenth tergite apically outwardly arcuate, basally only faintly inwardly arcuate. Spiculum gastrale (Figs 69–70) generally similar to that of *Exaesiopus grossipes*. Aedeagus (Figs 71–72) almost parallel-sided, apex with pseudopores, parameres fused along their apical half (approximately); aedeagus slightly curved ventrad from the lateral view (Fig. 72).

#### Differential diagnosis.

Generally the most punctate species of *Exaesiopus*, which can be confused only with densely punctate specimens of *Exaesiopus grossipes* from N Africa. It clearly differs from them by the punctation of pronotum as well as male genitalia (see also Key to species for details).

#### Biology.

Similar to that of other congeners – beetles are found in sand.

#### Distribution.

Kazakhstan; new to Uzbekistan and Russia.

### Exaesiopus atrovirens

Taxon classificationAnimaliaColeopteraHisteridae

Reichardt, 1926

Figs 73 , 74–79 , 80–88

Exaesiopus atrovirens Reichardt 1926: 17; Mazur (1984): 101; (1997): 263; (2004): 92; (2011): 210.

#### Type locality.

Arys, Kazakhstan.

#### Type material examined.

Holotype, ♂, with male genitalia extracted and glued to the subsequent label with male sign, with following labels: “♂” (printed); followed by circular golden label; followed by: “St. Arys / Tashkenskaya Zh d. / 27.v.[1]921 / na sklonach / saye, na osypyach / on the other side of the same label is written: “obryvystych kra- / yov i vypotov / soli. neredko / k reke / sb. I. Ivanov” (hand-written label on both sides); followed by: “Exaesiopus / atrovirens sp. n. / A. Reichardt det.” (written-printed label with black margin); followed by red, printed label: “Holotypus” (ZIN). Paratypes: 1 ♂ +1 ♀, with circular golden label, followed by written label: “St. Aris / Tashkenskaya Zh. d. / I. Ivanov 27.v.[1]921”; followed by: “Exaesiopus / atrovirens sp.n. / A. Reichardt det. (printed-written); followed by red label, written: “Paratypus”; followed by: “Zoological / Institute RAS / St. Petersburg” (yellow label, printed). 1 ♂, with circular golden label, followed by written label: “Askhabad”; followed by: “Exaesiopus / atrovirens sp. n. / A. Reichardt det.” (written-printed); followed by: “Paratypus” (hand-written red label); followed by: “Zoological / Institute RAS / St. Petersburg” (yellow label, printed); followed by yellow, pencil-written label: “09-060” (added by myself); 1 spec., with circular golden label, followed by: “Caucas, further illegible”; (black-turned, formerly red label, printed-written); followed by: “Coll. / Semenov Tian-Shansky” (written-printed); followed by: “Exaesiopus / atrovirens sp. n. / A. Reichardt det.” (written-printed label); followed by: “Paratypus” (hand-written red label); 1 ♂, with male genitalia extracted and glued to the subsequent label with male sign, with following labels: “♂” (printed); followed by circular golden label; followed by: “Caucasus / Coll. Kusnetzov / A. Semenov Tian-Shansky” (written-printed label); followed by: “Exaesiopus / atrovirens sp. n. / A. Reichardt det.” (written-printed label); followed by: “Paratypus” (hand-written red label); 1 spec., with circular golden label; followed by: “Owtshaly? / 11 mai / 1880” (written); followed by: “62” (pink label, written); followed by: “k. [=coll.] G. Siversa” (printed label in Russian); followed by: “Saprinus / grossipes / Mrs.” (written label); followed by: “Exaesiopus / atrovirens sp. n. / A. Reichardt det.” (written-printed label); followed by: “Paratypus” (hand-written red label); 1 spec., with golden circular label, followed by: “Turkestan / Sansar / Glasunov 1892” (black-margined printed label); followed by: “prope group / Hls. mit lang / Wimperhaaren / nur nicht / Akinini” (written); followed by: “k. [=coll.] A. Jacobsona” (printed); followed by: “Paratypus” (red label, written); 1 spec., with golden circular label, followed by: “St. Aris / Tashk. [Tashkentskaya] Zhe. [iron] d. [railway] / I. Uvarov 27.v.[1]921”; followed by: “Exaesiopus / atrovirens sp. n. / A. Reichardt det.” (written-printed label); followed by hand-written red label: “Paratypus”; followed by: “Zoological / Institute RAS / St. Petersburg” (yellow label, printed) (all type specimens ZIN).

#### Additional material examined.

Armenia: 1 spec., Yerevan, 19.v.1938, Richter (ZIN). Tajikistan: 1 ♀ & 1 spec., Pyandzh, from Khorog to Ishkashim, 6.vi.1928, Grishin leg. (ZIN); 1 spec., ibid, but BMNH. Azerbaijan: 1 spec., Khudat, SE Samura, 8.vii.1913, Lyukyanovitsh leg. (ZIN); Kazakhstan: 1 spec., Kazakhstan, river Ural near Kharkin, 7.v.1951, Gurjeva leg. (MNHN); 1 ♀, r. Ural near Kharkin, 13.v.1951, L. Arnoldi leg., under *Tamarix* in the sand (ZIN); 1 spec., Uralskaya obl., Kalmykov, 31.vii.1908, Borodin (ZIN); Russia: 2 specs., Volgogradskaya obl. Kamyshyn, 7.v.1939, Lyubyshev leg. (ZIN); 1 ♂, Dagestan, Terekli-Mekteb, Karanogaysk. steppe, 15.v.1925, Kirichenko leg. (ZIN); 5 specs., Astrakhan Region, Krasniy Yar district, near Dosang vill., 8.v.2009, A. Kovalyov leg. (CAS); 1 spec., ibid, 17.v.1998, 46°54'N, 47°54'E, K. Makarov & A.Brinyov leg (CAS). TURKEY: 1 spec., vill. Artvin, Cankurtaran Geç., 3.vi.2000, J. Mertlik leg. (TLAN); 1 spec., 21.vi.2003, Erzurum vill., ca 50 km S, Hamzalar - Hot Springs, 39°27'N, 41°07’, 1935 m, Jiří Hájek & Josef Hotový leg. (TLAN); 1 spec., Adana, 1903, no further data (BMNH). IRAN: Dorahi, 16.vi.1973, collector unknown (MNHN). Afghanistan: 1 ♂, Central, Gesab, 1400 m, 14.vi.1970, Kabakov leg. (ZIN). Georgia: 1 ♀, Mzcheta near Tbilisi, 12.–13.vi.1987, leg. Wrase & Schülke (NCB). UKRAINE: 1 spec., Kherson reg. Golaya Pristan distr. near Ribalche, 6.–9.v.1994, I. Melnik leg. (CAS).

#### Diagnostic description.

**Figure 73.**
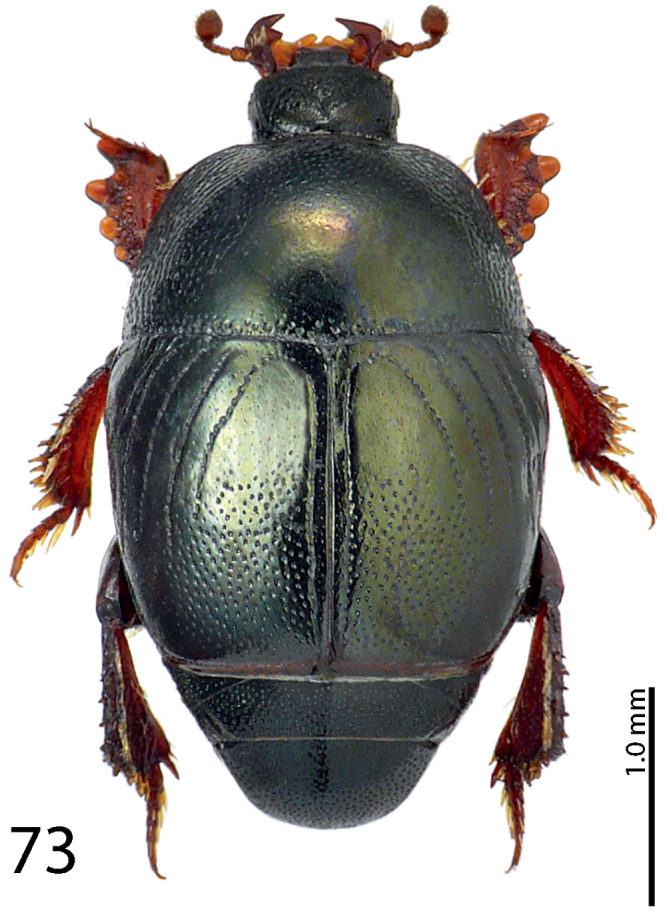
*Exaesiopus atrovirens* Reichardt, 1926 habitus.

**Figures 74–79.**
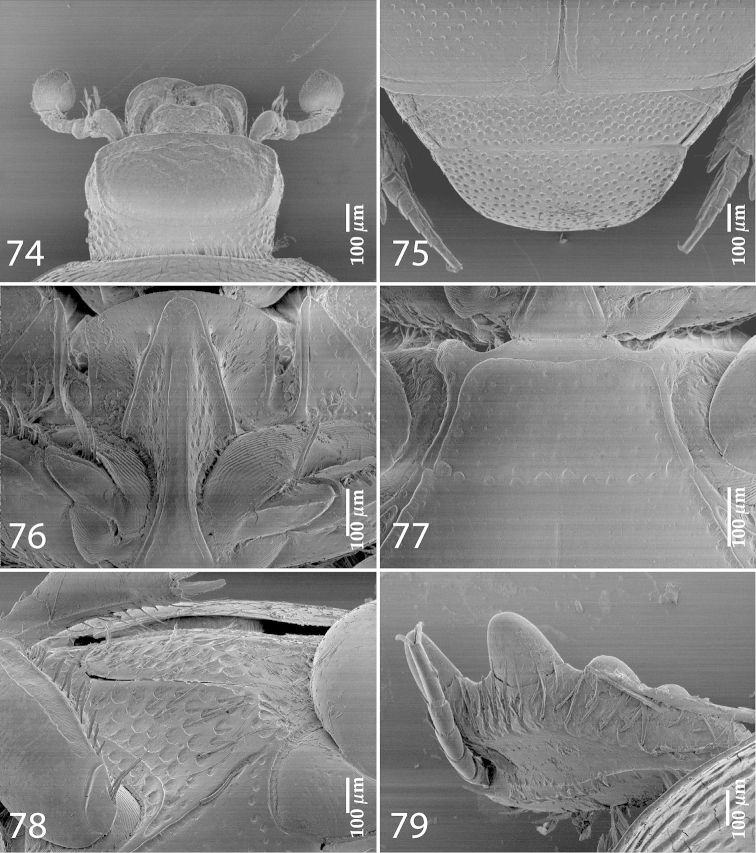
*Exaesiopus atrovirens* Reichardt, 1926 head, **74** dorsal view **75** propygidium + pygidium **76** prosternum **77** mesoventrite **78** lateral disc of metaventrite + metepisternum **79** protibia, dorsal view.

Body length: PEL: 2.50–2.75 mm; APW: 1.00–1.10 mm; PPW: 2.00–2.25 mm; EW: 2.125–2.40 mm; EL: 1.50–1.875. Body shape (Fig. 73) as in its congeners, cuticle with greenish metallic tinge; legs, mouthparts and antennae reddish-brown. Antennae as those of *Exaesiopus grossipes*; sensory structures of the antenna not examined. Mouthparts: mandibles somewhat more slender than those of *Exaesiopus grossipes*; labrum with large antero-median depression, otherwise similar to that of *Exaesiopus grossipes*; mentum and rest of the mouthparts likewise. Clypeus (Fig. 74) rectangular, rugose, anterior margin elevated, depressed medially; frontal, supraorbital and postorbital striae (Fig. 74) as in *Exaesiopus grossipes*; frons with several irregularly shaped carinate transverse rugae intermingled with numerous tiny rugae; at times transverse rugae obliterated under numerous tiny rugae; eyes flattened, but visible from above. Pronotum as in *Exaesiopus grossipes*. Elytra similar to that of *Exaesiopus grossipes*; inner subhumeral stria present medially; elytral punctation, however, mostly confined to apical half of elytra, only rarely punctures present on other than fourth elytral interval. Punctation of propygidium (Fig. 75) and pygidium similar to those of *Exaesiopus grossipes*, but punctures on propygidium almost confluent. Prosternum (Fig. 76) most similar to that of *Exaesiopus glaucus*, foveae small but deep; prosternal process asetose. Mesoventrite (Fig. 77) occasionally sparsely and finely punctate, otherwise similar to that of *Exaesiopus glaucus*; intercoxal disc of metaventrite similar to that of *Exaesiopus glaucus*; longitudinal depression in female very faint; lateral metaventral stria, rest of lateral disc of metaventrite, metepisternum + fused metepimeron (Fig. 78) most similar to those of *Exaesiopus glaucus*, but the amber setae distinctly longer and denser. Intercoxal disc of first abdominal sternite most similar to that of *Exaesiopus glaucus*. Protibia (Fig. 79) similar to that of *Exaesiopus glaucus*, differing from it chiefly by lower teeth topped by large triangular denticle. Mesotibia and metatibia similar to those of *Exaesiopus glaucus*; metatibia, however, slightly more thickened and dilated.

**Figures 80–88.**
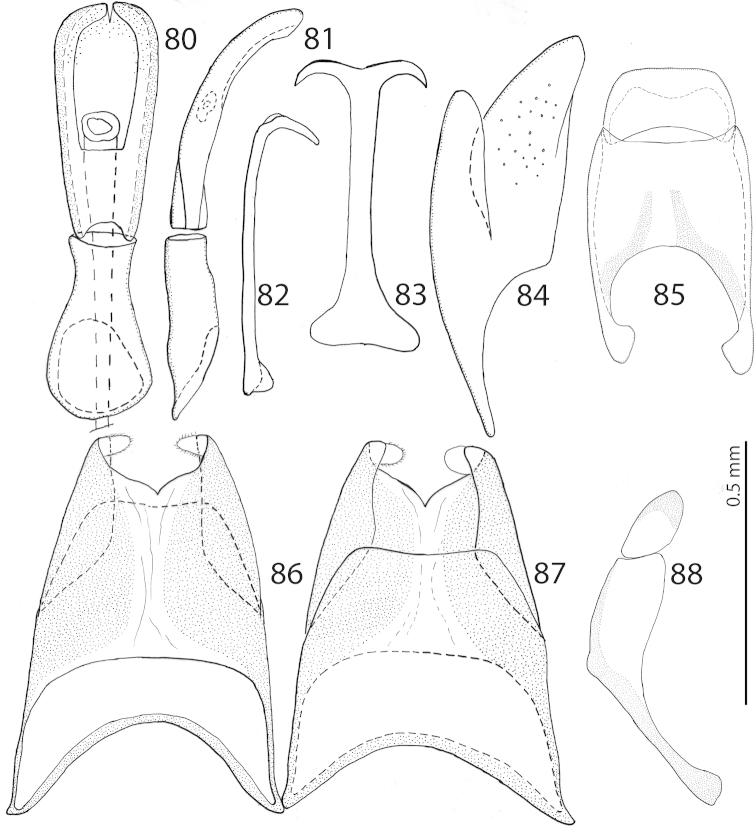
*Exaesiopus atrovirens* Reichardt, 1926 aedeagus, **80** dorsal view **81** ditto, lateral view **82** spiculum gastrale, lateral view **83** ditto, ventral view **84** 8^th^ sternite + tergite, lateral view **85** 9^th^ + 10^th^ tergites, dorsal view **86** 8^th^ sternite + tergite, ventral view **87** ditto, dorsal view **88** 9^th^ + 10^th^ tergites, lateral view.

Male genitalia. Eighth sternite (Fig. 86–87) fused along its entire length, apically asetose, velum tiny; eighth tergite apically faintly inwardly arcuate; eighth sternite and tergite fused laterally (Fig. 84). Ninth tergite (Figs 85, 88) apically faintly inwardly arcuate; spiculum gastrale (Figs 82–83) similar to other congeners. Aedeagus (Figs 80–81) short and stout, gradually dilated anteriorly, apex with pseudopores, parameres fused along their basal two-thirds (approximately), aedeagus slightly curved ventrad (Fig. 81).

#### Differential diagnosis.

*Exaesiopus atrovirens* is most similar externally to *Exaesiopus glaucus*, differing from it by longer vestiture on underside of the body, numerous irregular rugae of frons, more thickened and dilated metatibia, larger triangular denticles of protibia, and male genitalia (compare Figs 80–88 with Figs 107–113; see also Key to species for details). From the rest of the congeners it differs chiefly by its greenish metallic hue of the dorsum (other species are not metallic).

#### Biology.

Found in sand, often under *Tamarix*.

#### Distribution.

Known from Turkey, Russia, Armenia, Azerbaijan, Georgia, Kazakhstan, Iran, Afghanistan and Turkmenistan. New to Ukraine and Tajikistan.

### Exaesiopus laevis

Taxon classificationAnimaliaColeopteraHisteridae

Thérond, 1964

Figs 89 , 90–97

Exaesiopus laevis Thérond 1964: (3) 72; Mazur (1984): 101; Mazur (1997): 264; Mazur (2011): 210.

#### Type locality.

Guardafui, Somalia.

#### Type material examined.

Holotype, ♀, mounted on its side on a triangular point, right protibia missing, with printed label: “SOMALI REP. / North region”, followed by another printed label: “Guardafui / XI. 1959 / C. Hemming”; with another printed-written label: “J. Thérond det., 1962 / Exaesiopus / laevis n. sp.” and a red label attached to it (printed-written): “TYPE / Esemplare / unico”; with another yellow, pencil-written label: “D08-092”, added by myself (MSNM).

#### Diagnostic description.

Body length: PEL: 2.375 mm; APW: 0.825 mm; PPW: 1.75 mm; EL: 1.50 mm; EW: 2.00 mm.

**Figure 89.**
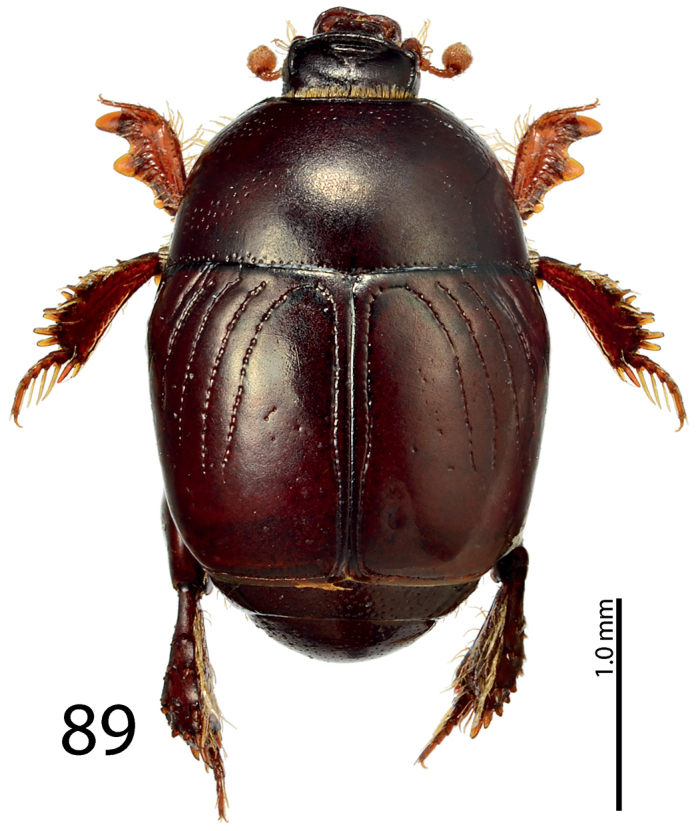
*Exaesiopus laevis* Thérond, 1964 holotype, habitus.

**Figures 90–97.**
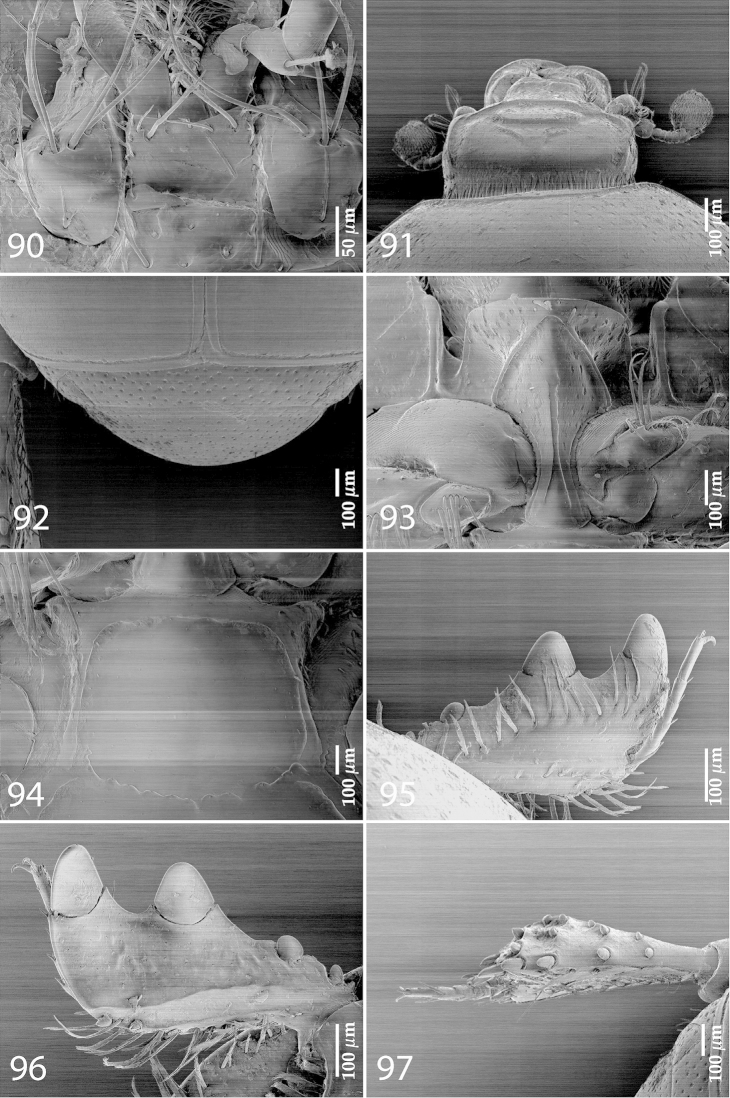
*Exaesiopus laevis* Thérond, 1964 holotype, mentum, **90** ventral view **91** holotype, head, dorsal view **92** holotype, propygidium + pygidium **93** holotype, prosternum **94** holotype, mesoventrite **95** holotype, protibia, dorsal view **96** ditto, ventral view **97** holotype, metatibia, dorsal view.

Body (Fig. 89) without metallic tinge; legs, mouthparts and antennae light brown; antennal club amber. Antennae as in *Exaesiopus grossipes*; sensory structures of the antennal club not examined. Mouthparts: mentum (Fig. 90) glabrous, sub-quadrate, shallowly inwardly arcuate on anterior margin; anterior margin with several rather long setae intermingled with short sparse ramose setae; rest of the mouthparts as in *Exaesiopus grossipes*. Clypeus and frons (Fig. 91) similar to those of *Exaesiopus henoni*. Pronotum almost smooth, only laterally and behind head with vague patches of shallow sparse punctation; otherwise similar to that of *Exaesiopus henoni*. Elytra: inner subhumeral stria absent; elytral disc entirely smooth. Propygidium and pygidium (Fig. 92) similar to other congeners, but only sparsely punctate, punctures separated by several times their own diameter. Prosternum (Fig. 93): prosternal foveae tiny, almost invisible; prosternal process otherwise similar to that of other congeners. Mesoventrite (Fig. 94) glabrous, about as long as wide; metaventrite smooth; lateral disc of metaventrite and metepisternum similar to those of *Exaesiopus henoni*. Intercoxal disc of first abdominal sternite similar to that of *Exaesiopus henoni*. Protibia (Figs 95–96) similar to that of *Exaesiopus henoni*, but outer margin of teeth topped by large triangular denticles, more similar in size than those of *Exaesiopus henoni*, furthermore outer part of posterior surface of protibia of *Exaesiopus laevis* obscurely variolate, whereas it is glabrous in *Exaesiopus henoni*. Mesotibia generally similar to that of *Exaesiopus henoni*, but denticles on outer margin shorter. Metatibia (Fig. 97) likewise generally similar to that of *Exaesiopus henoni*, but denticles on outer margin more numerous.

Male unavailable.

#### Differential diagnosis.

This species is most similar to *Exaesiopus henoni*, from which it differs by almost impunctate pronotum (punctate in *Exaesiopus henoni*), smooth elytra (punctate in *Exaesiopus henoni*) and obscurely variolate posterior surface of protibia (glabrous in *Exaesiopus henoni*). From the rest of *Exaesiopus* species it differs by the characters given in the Key to species (below).

#### Biology.

Unknown, possibly similar to the congeners.

#### Distribution.

Known only from north-extreme tip of Somalia: Guardafoui.

#### Remarks.

This species is morphologically rather similar to *Exaesiopus henoni*, which is known also from the neighbouring Djibouti. The discovery of a male of *Exaesiopus laevis* would help to elucidate the identities of the two respective species.

### Exaesiopus glaucus

Taxon classificationAnimaliaColeopteraHisteridae

(Bickhardt, 1914) comb. n.

Figs 98 , 99–106 , 107–113

Pachylopus glaucus Bickhardt 1914: 280.
Hypocaccus (Hypocaccus) glaucus : Mazur (1984): 94; Mazur (1997): 259; Mazur (2011): 206.

#### Type locality.

Gobabeb, Namibia.

#### Type material examined.

Neotype, ♂, side-mounted on a triangular mounting point, right antennal club broken off, both terminal metatarsomeres broken off, with male genitalia mounted in Canada balsam on a separate slide under specimen, with the following labels: “S.W. Afr., Namib / Gobabeb / 23.34S–15.03E” (printed); followed by: “24.9.1974; E–Y: 376 / shore washing / leg. Endrödy-Younga” (printed); followed by: “Exaesiopus / glaucus / Bickh. / det. J. Thérond” (printed-written); followed by: “D08-029” (yellow, pencil-written label, written by myself); followed by: “Pachylopus glaucus / Bickhardt, 1914 / NEOTYPE det. T. / Lackner 2014” (red label, written) (TMSA).

#### Note.

This species has been described based on a single specimen collected in Okahandja (Namibia) (Bickhardt 1914: 280). According to the personal information by the curator of ZMHUB B. Jaeger, the specimen was deposited at the Hamburg Museum of Natural History (Germany), which has been destroyed during WWII. The type specimen of this species can thus be considered as lost and hence a Neotype is designated herein.

#### Additional material examined.

NAMIBIA: 1 ♂ + 1 ♀, Gobabeb, 23.34S – 15.03E, 24.ix.1974, Endrödy-Younga leg., shore-washing (TMSA); 1 ♂ + 1 ♀, ibid, but MNHN; 1 ♀, Swakop River, 3 miles S of Okahandja, 7.iv.1972, floating refuse (MNHN). REPUBLIC OF SOUTH AFRICA: 1 ♂, Cape-Cedarbg, Olifants R., Boshof, 32.20S – 18.59E, 20.viii.1983, Endrödy-Younga & Penrith leg., sand banks, river (TMSA).

#### Diagnostic description.

**Figure 98.**
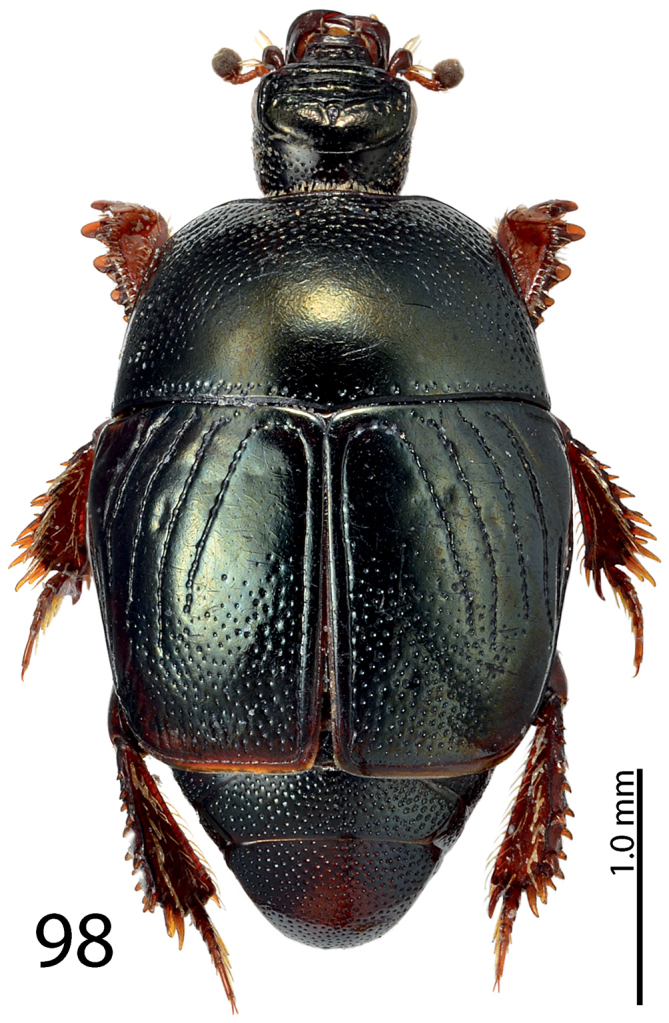
*Exaesiopus glaucus* (Bickhardt, 1914) habitus.

**Figure 99–106.**
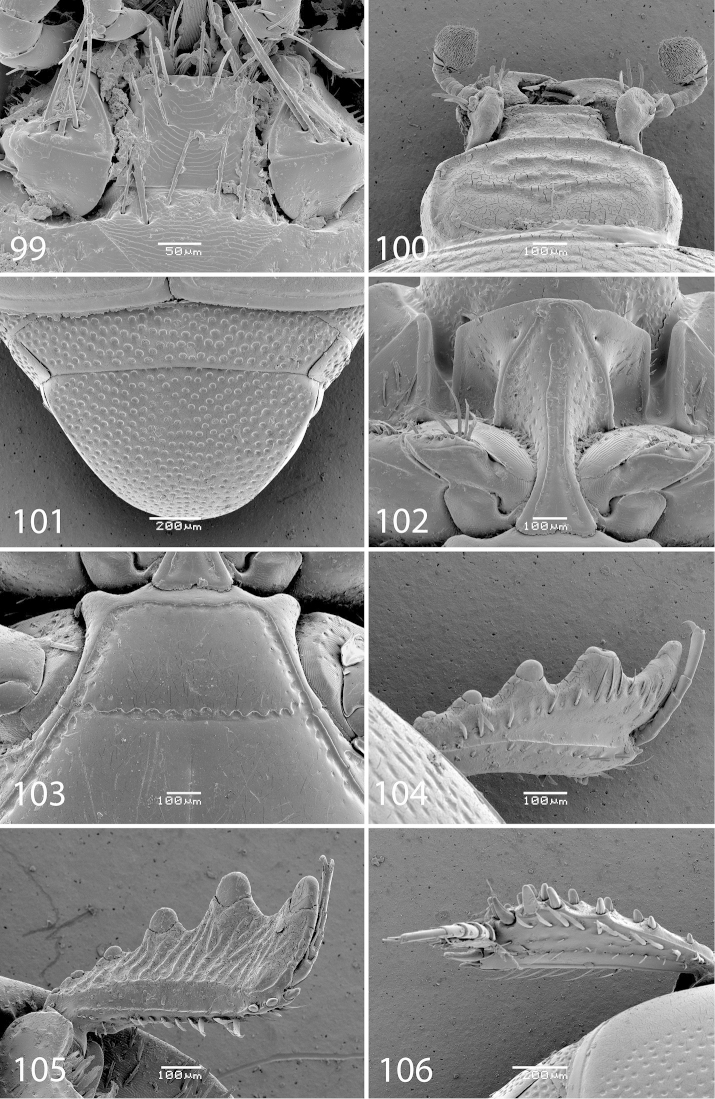
*Exaesiopus glaucus* (Bickhardt, 1914) mentum, **99** ventral view **100** head, dorsal view **101** propygidium + pygidium **102** prosternum **103** mesoventrite **104** protibia, dorsal view **105** ditto, ventral view **106** metatibia, dorsal view.

**Figures 107–113.**
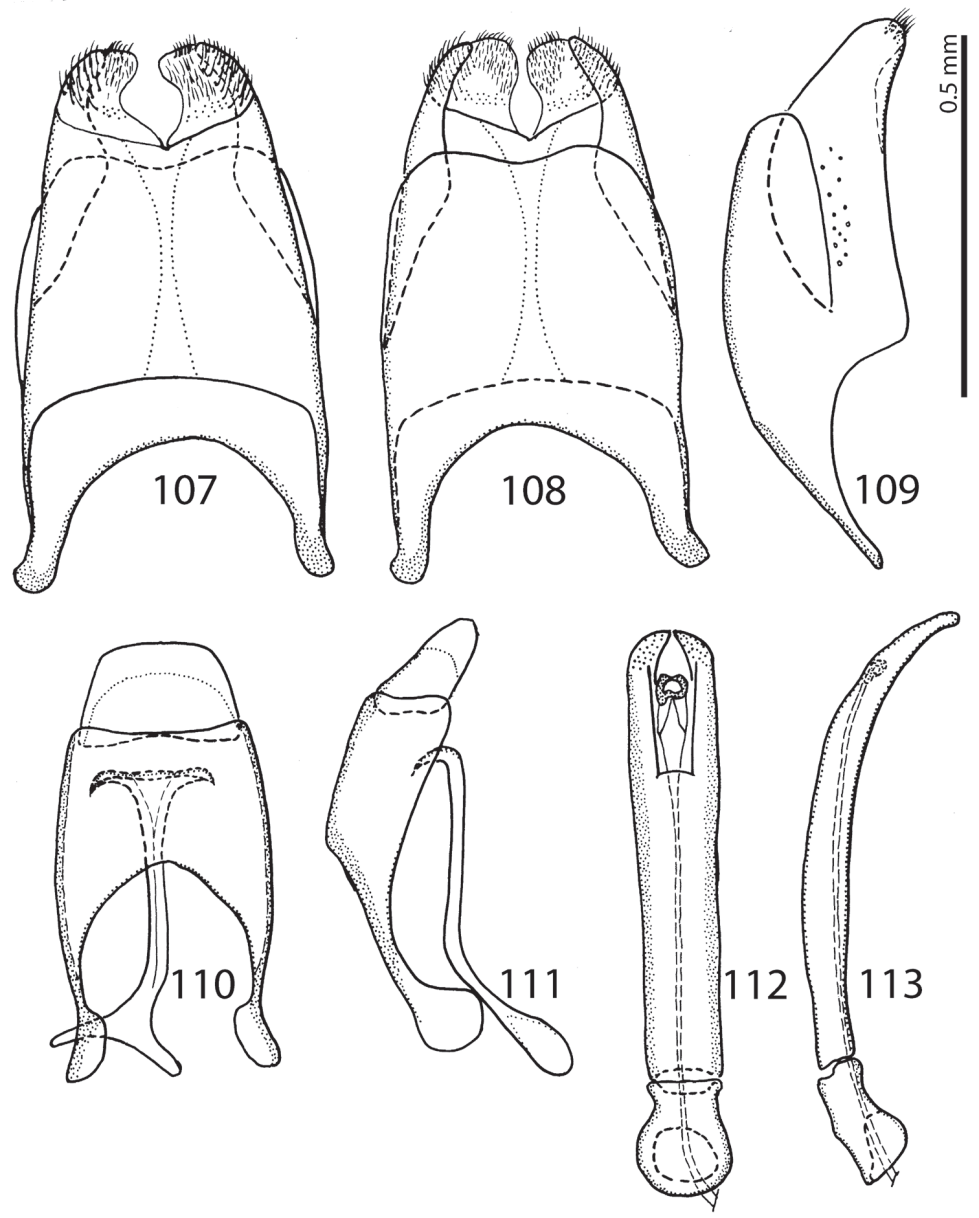
*Exaesiopus glaucus* (Bickhardt, 1914) 8^th^ sternite + tergite, **107** ventral view **108** ditto, dorsal view **109** ditto, lateral view **110** 9^th^ + 10^th^ tergites dorsal view & spiculum gastrale, ventral view **111** 9^th^ + 10^th^ tergites & spiculum gastrale, lateral view **112** aedeagus, dorsal view **113** ditto, lateral view.

Body length: PEL: 2.50–2.60 mm; APW: 0.80–1.00 mm; PPW: 1.83–2.00 mm; EW: 2.00–2.18 mm; EL: 1.50–1.60 mm. Body (Fig. 98) similar to the species *Exaesiopus atrovirens*, with feeble metallic tinge; legs, mouthparts and antennae light brown. Antennae as in *Exaesiopus grossipes*. Mouthparts: as in *Exaesiopus grossipes*; labrum with median keel-like elevation, surface anterad of it semi-circularly depressed; mentum (Fig. 99) sub-trapezoid, anterior margin without median notch, fringed with several long setae, lateral margins with single row of sparse shorter ramose setae; stipes with four setae; other mouthparts similar to those of *Exaesiopus atrovirens*. Clypeus (Fig. 100) rectangular, obscurely variolate, anterior margin elevated, formed by two transverse tubercles that can occasionally be connected forming thus a ridge-like structure; clypeus and frons otherwise similar to those of *Exaesiopus atrovirens*, but without numerous irregular rugae. Pronotum: sides slightly convergent on basal 3/4, strongly convergent on apical ¼; disc with round dense punctation, laterally punctures larger in size and increasingly ellipsoid, occasionally confluent; postero-median part of disc smooth, punctation stops short of lateral pronotal margin leaving a narrow impunctate band; pronotal base with a single row of round punctures; pronotal hypomeron with short amber setae almost invisible from dorsal view; scutellum small, visible. Elytra: humeral elytral stria well impressed on basal fourth; inner subhumeral stria present medially as a short median fragment; elytral punctation confined to apical half of elytra, along elytral suture reaches up to 2/3 of elytral length anteriorly, punctures in most cases do not enter elytral intervals, regular and deep, separated by about their own diameter, punctation does not become denser apically; rest of elytra impunctate. Propygidium and pygidium (Fig. 101) similar to other congeners, with coarse and dense regular punctation. Prosternum (Fig. 102): prosternal foveae well impressed, rather small, but deep; prosternal process slightly concave, otherwise similar to that of other congeners. Mesoventrite (Fig. 103) slightly wider than long, almost smooth; meso-metaventral sutural stria well impressed, undulate; intercoxal disc of metaventrite with longitudinal depression in both sexes, more prominent in male, almost smooth, except for several rows of variously-sized deep punctures along base; lateral metaventral stria, lateral disc of metaventrite and metepisternum similar to those of *Exaesiopus henoni*. Intercoxal disc of first abdominal sternite as with the rest of congeners. Protibia (Fig. 104) on outer margin with two moderately large triangular teeth, topped by rounded denticle followed by another two lower teeth topped by small round denticle and another tiny denticle entombed in outer protibial margin; setae of outer row regular and short; setae of median row shorter than those of outer row; anterior protibial stria almost complete; protibial groove deep; protibial spur (Fig. 105) distinct but tiny, growing out from apical margin of protibia; outer part of posterior surface of protibia rugulose-lacunose, clearly separated from comparatively narrower glabrous median part; posterior protibial stria complete, terminating in two tiny inner posterior denticles; inner margin of protibia with single row of short lamellate setae. Mesotibia not particularly dilated or thickened, outer margin similar to that of *Exaesiopus henoni*; posterior mesotibial stria fine, shortened apically; mesotibial spur stout, prominent and long; anterior surface of mesotibia smooth; anterior mesotibial stria shortened apically; claws of last tarsomere almost straight, their length approximately half the length of apical-most mesotarsomere. Metatibia (Fig. 106) slightly more dilated and thickened than mesotibia, but always more slender than that of the rest of the congeners; two rows of denticles on outer margin widely separated permitting for placement of another two denticles between the two rows; claws of apical-most metatarsomere shorter than half its length; otherwise metatibia similar to mesotibia. Male genitalia. Eighth sternite (Figs 107–108) entirely fused medially, apically with a setose velum; apex of eighth sternite with short dense setae. Eighth tergite apically weakly inwardly arcuate; eighth sternite and tergite fused laterally (Fig. 109). Ninth tergite (Fig. 110) on apical margin faintly inwardly arcuate; tenth tergite on apical margin regularly rounded, weakly inwardly arcuate basally. Spiculum gastrale (Figs 110–111) with typical ‘head’ and ‘tail’; aedeagus (Figs 112–113) tube-like, sub-parallel, slightly widening apically; parameres fused along their basal three-fourths, apex of aedeagus with pores; basal piece short, ratio of its length : length of parameres approximately 1:5.

#### Differential diagnosis.

*Exaesiopus glaucus* is arguably the most distinctive species of the genus differing from all other members by only slightly dilated metatibia (strongly dilated in all other species, compare Fig. 106 with e.g. 97); present and observable protibial spur (very tiny or absent in the rest of species, compare Fig. 105 with e.g. 43). Furthermore, the setae of the pronotal hypomeron are rather short and invisible from dorsal view (in all other species they are protruding from underside of the pronotum and are observable from dorsal view).

#### Biology.

Found on a beach by the technique of shore-washing as well as on a river bank on deposited debris.

#### Distribution.

Described from Namibia; newly recorded from the Republic of South Africa.

#### Remarks.

The placement of this species in *Exaesiopus* must be regarded as tentative, as it differs from the rest of the members chiefly by only slightly instead of strongly dilated metatibiae. *Hypocaccus* from the Old World, however, does not contain any species with ciliate pronotal hypomera, and keeping *Exaesiopus glaucus* in *Hypocaccus* would make it heterogeneous. Note that it was already Reichardt (1926) who remarked that this species should be, based on its ciliate pronotal hypomeron, moved into the genus *Exaesiopus*. Thérond, in the 1960’s and 1970’s identified this species as ‘*Exaesiopus*’, rather than ‘*Hypocaccus*’ *glaucus*.

### Exaesiopus therondi sp. n.

Taxon classificationAnimaliaColeopteraHisteridae

http://zoobank.org/E2EBDF60-6401-43CB-B106-3EF5E926E3EF

Figs 114 , 115 , 116 , 117 , 118–126

#### Type locality.

Hamud-i-Sabari, Afghanistan.

#### Type material examined.

Holotype, ♂, side-mounted on a triangular point, right hind leg missing, genitalia glued to the same mounting point as the specimen, with the following labels: “N AFGHANISTAN: / Hamud-i-Sabari / 26.iii.1949 Danish / Central Asian Expedn.” (written in black ink); followed by: “Pachylopus / sp. not in BM / J. Balfour-Browne det. / v. 1964” (written-printed); followed by: “St. No. / 7” (printed-written); followed by: “Brit. Mus. / 1964-302” (printed-written); followed by: “Ex stomach of / Charadinus alexandrinus / alexandrinus L.” (written in black ink); followed by: “Exaesiopus / n. sp. ? / J. Thérond det. 1964” (written-printed); followed by: “Exaesiopus / therondi n.sp. / HOLOTYPE / det. T. Lackner 2014” (red label, written) (BMNH).

#### Diagnostic description.

**Figure 114.**
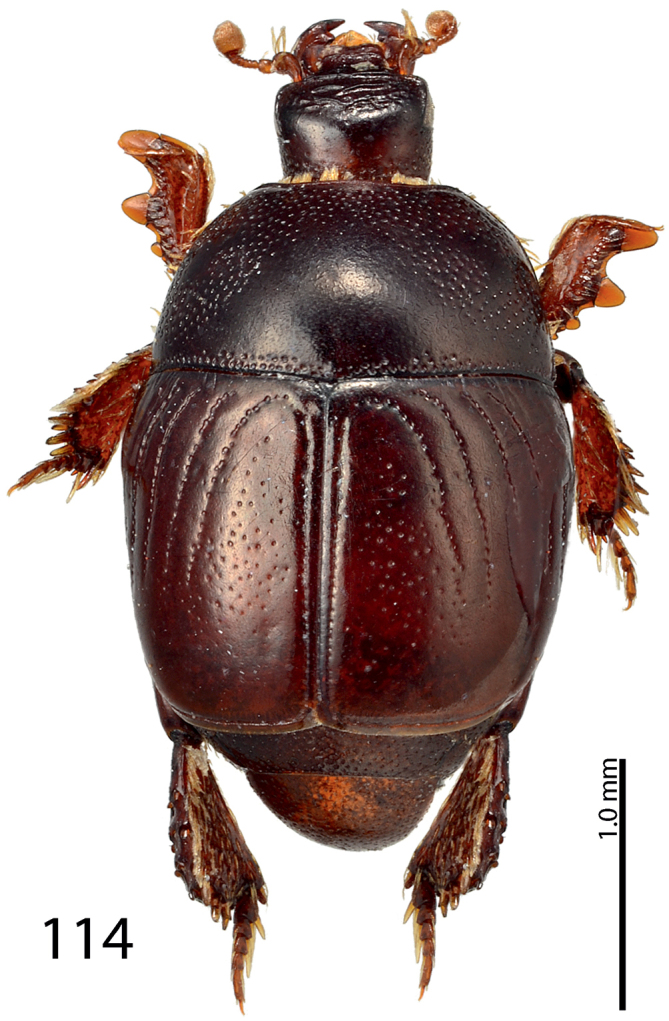
*Exaesiopus therondi* sp. n. habitus, dorsal view.

**Figure 115.**
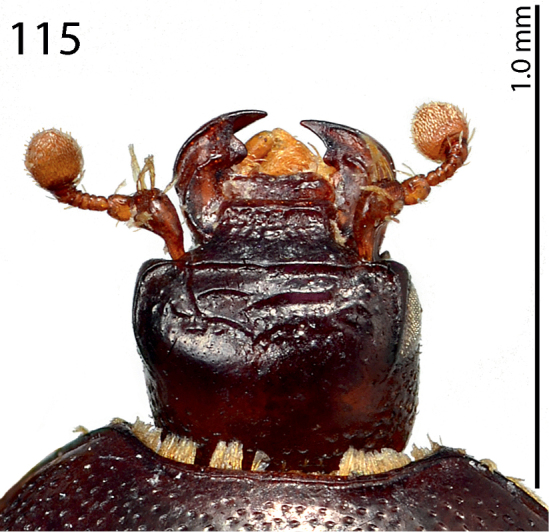
*Exaesiopus therondi* sp. n. head, dorsal view.

**Figure 116.**
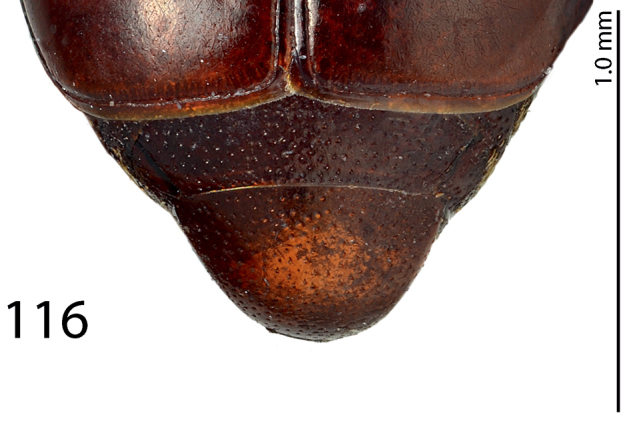
*Exaesiopus therondi* sp. n. propygidium + pygidium.

**Figure 117.**
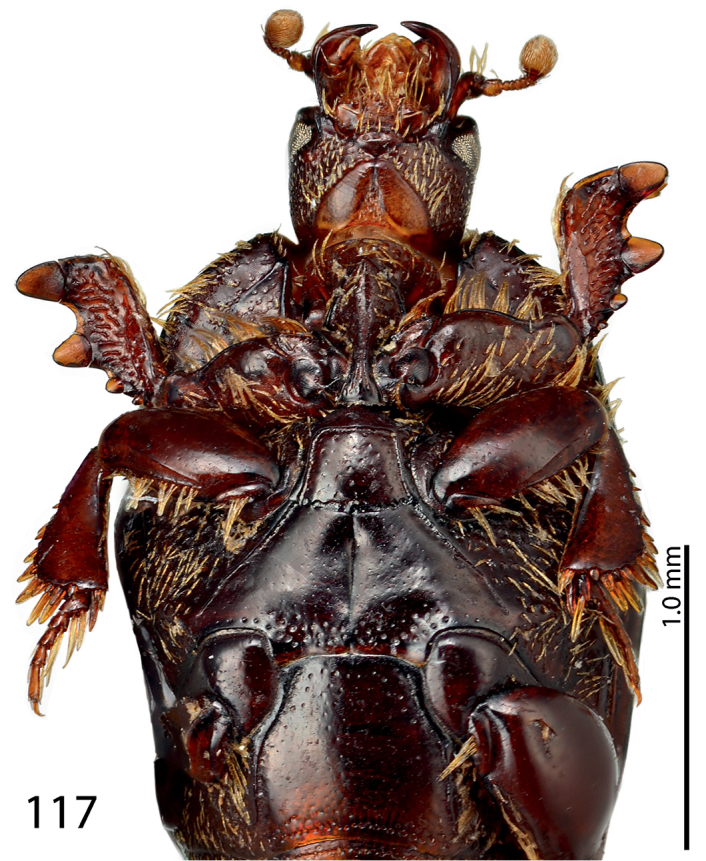
*Exaesiopus therondi* sp. n. habitus, ventral view.

**Figure 118–126.**
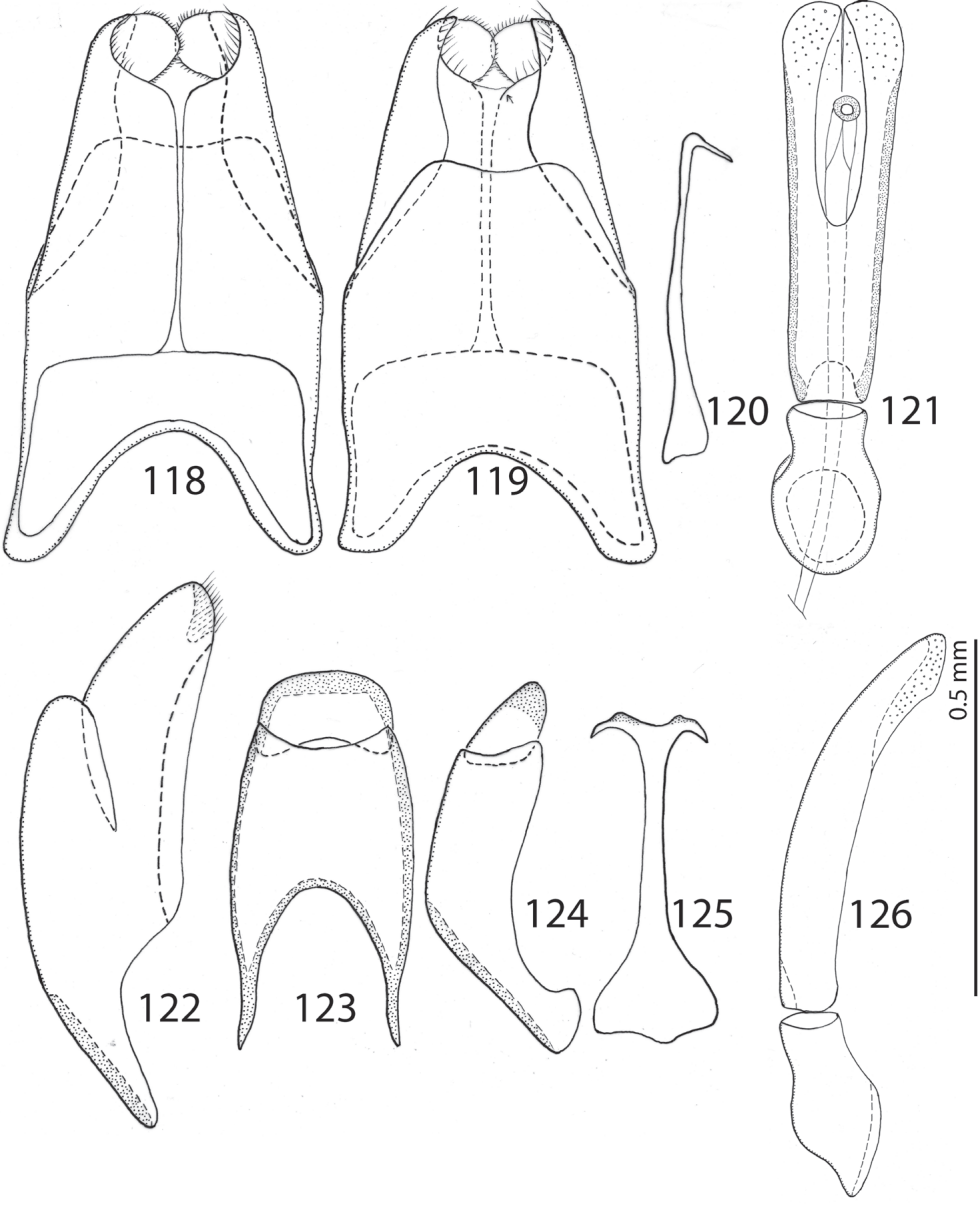
*Exaesiopus therondi* sp. n. 8^th^ sternite + tergite, **118** ventral view **119** ditto, dorsal view **120** spiculum gastrale, lateral view **121** aedeagus, dorsal view **122** 8^th^ sternite + tergite, lateral view **123** 9^th^ + 10^th^ tergites, dorsal view **124** ditto, lateral view **125** spiculum gastrale, ventral view **126** aedeagus, lateral view.

Body length: PEL: 2.125 mm; APW: 0.875 mm; PPW: 1.825 mm; EW: 2.05 mm; EL: 1.55 mm. This species (Fig. 114) is externally very similar to *Exaesiopus henoni*, differing from it chiefly by its densely punctate pronotum, which is furnished with two round glabrous patches amongst the punctation laterally. The structure of frons (Fig. 115) is also different; whereas *Exaesiopus henoni* always possesses only two well-defined chevrons on a completely glabrous surface, *Exaesiopus therondi* has its chevrons beset on all sides with irregular rugae. The punctation of propygidium and pygidium (Fig. 116) is similar to that of *Exaesiopus henoni* (Fig. 38). The prosternal process (Fig. 117) of *Exaesiopus therondi* is more setose than that of *Exaesiopus henoni*; prosternal foveae are absent. Anterior face of profemora (Fig. 117) is covered with dense amber setae in *Exaesiopus therondi*, whereas only several sparse short setae are present in *Exaesiopus henoni*. Anterior face of protibia (Fig. 117) is rugulose-lacunose in *Exaesiopus therondi* while it is glabrous in *Exaesiopus henoni*. Further differences are found on male genitalia: Eighth sternite (Figs 118–119) is more slender, setae on apex are shorter; eighth sternite and tergite apically more slender (seen from lateral view; compare Figs 48 and 122). The rest of the male genitalia is markedly similar between the two species.

#### Differential diagnosis.

*Exaesiopus therondi* most resembles the Saharan species *Exaesiopus henoni*, differing from it by rugulose-lacunose anterior face of protibia (glabrous in *Exaesiopus henoni*), and the different structure of the frons (*Exaesiopus henoni* has its frons glabrous with two chevrons whereas *Exaesiopus therondi* has the chevrons surrounded by tiny rugae).

#### Biology.

Unknown, found in a stomach of Kentish plover (*Charadrius alexandrinus* L.).

#### Distribution.

Known only from Afghanistan: Hamud-i-Sabari.

#### Remarks.

Although this newly described species does strongly resemble the Saharan species *Exaesiopus henoni*, and it has furthermore been found in a stomach of a bird, it is unlikely that they are conspecific, given the vast geographic stretch between African Sahara and Afghanistan. If it had been consumed by a Kentish plover in Africa and discovered in its stomach in Afghanistan it would have probably passed through the digestive tract of the bird by the time the bird migrated from the Sahara Desert to Afghanistan and would be beneath recognition at best. Instead, given the perfect shape of the insect, I consider it highly probable that the bird consumed it in Afghanistan and thus this species is an element of the Afghan fauna.

### Key to the species of the genus *Exaesiopus* Reichardt, 1926

**Table:** 

1 (2)	Mesotibia only slightly thickened and dilated (Fig. 106); protibial spur distinct (Fig. 105); species from Namibia and the Republic of South Africa	***Exaesiopus glaucus* (Bickhardt, 1914), comb. n.**
2 (1)	Mesotibia strongly dilated and thickened (Fig. 97); protibial spur tiny, not easily observable to outright absent (Fig. 96), species from the Palaearctic Region or Horn of Africa.	
3 (8)	Protibia with two large teeth topped by large triangular denticle followed by one to three tiny denticles entombed in outer protibial margin (Figs 43, 96, 117).	
4 (5)	Anterior face of protibia (Fig. 43) glabrous; prosternal foveae (Fig. 39) inconspicuous (absent?); species from the Sahara (Morocco, Algeria, Libya) and Djibouti	***Exaesiopus henoni* (Schmidt, 1896)**
5 (4)	Anterior face of protibia (Figs 96, 117) obscurely variolate to rugulose lacunose; prosternal foveae tiny, but observable (Fig. 93); species from Somalia and Afghanistan.	
6 (7)	Almost completely glabrous species, with scattered faint punctation on pronotum only (Fig. 89); inner subhumeral stria absent; frons (Fig. 91) with two well-marked chevrons without additional rugae, species from extreme northern tip of Somalia	***Exaesiopus laevis* Thérond, 1964**
7 (6)	Punctate species (Fig. 114); frons (Fig. 115) except for chevrons also with additional tiny rugae, species from Afghanistan	***Exaesiopus therondi* sp. n.**
8 (3)	Protibia on outer margin with two to three low teeth topped by large triangular or rounded denticles, followed by two to three lower rounded denticles entombed in outer protibial margin (Figs 10, 61, 79).	
9 (10)	Elytral punctation mostly confined to apical third to half of elytra, never occupying all elytral intervals; species with feeble to distinct green metallic hue (Fig. 73)	***Exaesiopus atrovirens* Reichardt, 1926**
10 (9)	Elytral punctation occasionally entering elytral intervals, in extreme cases covering entire elytral disc (Fig. 55); species without metallic hue.	
11 (12)	Punctation of pronotum reaches pronotal margin, covering almost entire pronotal disc (Fig. 55); male genitalia: eighth tergite and sternite slightly more dilated than in the following species; aedeagus parallel-sided (Figs 64–72), species from S Russia and Middle Asia (Kazakhstan, Uzbekistan)	***Exaesiopus torvus* Reichardt, 1926**
12 (11)	Punctation of pronotum does not reach pronotal margin, leaving antero-median part of pronotum glabrous (Fig. 1); male genitalia: eighth sternite and tergite slightly more slender than in the preceding species; aedeagus on apical half slightly thickened (Figs 17–34), species from the circum-Mediterranean, Canary Islands, S Europe and Iraq	***Exaesiopus grossipes* (Marseul, 1855)**

## Discussion

*Exaesiopus* is a taxon that is morphologically well adapted to the psammophilous and fossorial way of life by the thickened metafemora as well as dilated pro- and especially metatibiae. A setose underside of the body is common to most obligate psammophiles in Histeridae and serves as further adaptation to life in sand; setae possibly prevent tiny particles of sand entering the body cavities. Although morphologically united by at least one weak synapomorphy (ciliate pronotal hypomeron), which is possibly a parallelism shared by some *Hypocaccus* spp. from North America, the monophyly of the genus *Exaesiopus* is likely questionable. The taxonomical uncertainties between (mostly) littoral taxa *Hypocaccus*, *Exaesiopus*, *Pachylopus*, *Neopachylopus*, *Eopachylopus*, etc. lie chiefly in the morphological similarities resulting from ecological pressures causing multiple parallelisms and convergences of characters. A future phylogenetic analysis of all littoral *Hypocaccus*-like taxa should focus on characters in systems putatively independent of the environmental selection pressures; otherwise characters that are prone to homoplasies (e.g. setae, denticles, rugae, trichomes etc.) could continue to obscure true phylogenetic relationships. In the recently published phylogeny of the subfamily by the author (Lackner 2014d), which included mostly the type species of the Saprininae genera, the type species of *Exaesiopus* was recovered among the members of a large clade of mostly psammophilous taxa whose inter-relationships are unresolved.

Members of *Exaesiopus* are found in sandy soils or in sand over a vast geographic area rivalling perhaps only the distribution of *Xenonychus* Wollaston, 1864 (see also Lackner 2012). The distribution of *Exaesiopus* covers the area from the Canary Islands, circum-Mediterranean, South Europe, Caucasus, Iraq, Somalia, Djibouti, as far east as Afghanistan. Identity of the Somali species *Exaesiopus laevis* Thérond, 1964 is uncertain; the species is known from a single female only. Other related genera (sensu Lackner, 2014) e.g. the species-rich and widespread *Hypocaccus* or *Hypocacculus*, or monotypic and localized *Eopachylopus*, *Reichardtia* etc., are distributed along most of the world beaches, as well as inland sand-systems; their inter-relationships shall be the focus of future phylogenetic studies.

## Supplementary Material

XML Treatment for Exaesiopus

XML Treatment for Exaesiopus grossipes

XML Treatment for Exaesiopus henoni

XML Treatment for Exaesiopus torvus

XML Treatment for Exaesiopus atrovirens

XML Treatment for Exaesiopus laevis

XML Treatment for Exaesiopus glaucus

XML Treatment for Exaesiopus therondi
